# *VqMYB154* promotes polygene expression and enhances resistance to pathogens in Chinese wild grapevine

**DOI:** 10.1038/s41438-021-00585-0

**Published:** 2021-07-01

**Authors:** Changyue Jiang, Dan Wang, Jie Zhang, Yan Xu, Chaohong Zhang, Jianxia Zhang, Xiping Wang, Yuejin Wang

**Affiliations:** 1 College of Horticulture, Northwest A & F University, 712100 Yangling, Shaanxi The People’s Republic of China; 2 Key Laboratory of Horticultural Plant Biology and Germplasm Innovation in Northwest China, Ministry of Agriculture, 712100 Yangling, Shaanxi The People’s Republic of China; 3 State Key Laboratory of Crop Stress Biology in Arid Areas, Northwest A & F University, 712100 Yangling, Shaanxi The People’s Republic of China

**Keywords:** Plant signalling, Biotic, Microbe, Transcriptional regulatory elements

## Abstract

Resveratrol plays a crucial phytoalexin role in the grapevine and is beneficial to human health. However, the molecular mechanism of resveratrol accumulation in the enhancement of disease resistance is unclear. Here, we report that the transcription factor VqMYB154 from *Vitis quinquangularis* accession Danfeng-2 is strongly expressed under artificial inoculation with *Uncinula necator* and regulates resveratrol accumulation. Unlike its homolog, *VqMYB154* has a pathogen-induced promoter and responds to stimulation by *U. necator*, *Pseudomonas syringae*, and other treatments. Yeast one-hybrid and GUS activity assays confirmed that VqMYB154 can activate the stilbene synthase genes *VqSTS9*, *VqSTS32*, and *VqSTS42* by directly binding to their promoters. Overexpression of *VqMYB154* in grape leaves resulted in activation of the stilbene pathway, upregulation of *STS* genes, and accumulation of stilbenoids. In addition, heterologous overexpression of *VqMYB154* in *Arabidopsis* activated resistance-related genes and resulted in greater programmed cell death and accumulation of reactive oxygen species, which led to resistance against *P. syringae*. These results suggest that the transcription factor VqMYB154 from *V. quinquangularis* accession Danfeng-2 participates in the regulatory mechanism that improves the biosynthesis and accumulation of stilbenes and enhances resistance to disease in grapevine.

## Introduction

Grapevine is one of the most prestigious economic fruit crops worldwide. According to data from the Food and Agriculture Organization, the total output of grapes ranked third among fruit crops in 2018. Of *Vitis vinifera* grown worldwide, few cultivars possess high resistance to phytopathogenic microorganisms, including *Uncinula necator*^1^. If left unchecked, pathogen-triggered diseases will seriously affect the growth and quality of grapevine, ultimately leading to a decline in yield. Long-term use of pesticides pollutes the environment, and hazardous residues in grape berries threaten human health^2^. Therefore, it is crucial to improve grapevine resistance to reduce the need for pesticide application. Some resistant grape species, such as *V. labrusca*, have an unacceptable foxy smell, which greatly limits their application in crossbreeding^3^. China is a vital area of origin for wild grapevine germplasm, with many disease-resistant *Vitis* spp^4^. These wild species do not have undesirable flavors, and can easily be crossed with *V. vinifera* cultivars^5^; thus, they provide critical resources for resistance breeding of grapevine.

As a well-known stilbene-producing plant, the grapevine has high levels of resveratrol. This bioactive compound was first extracted from *Veratrum grandiflorum*^6^ and was determined to be present in grape tissues^7^. Resveratrol has great research value. Furthermore, it is beneficial to human health due to its antioxidant activity^8^ and antiaging^9^, neuroprotective^10^, and cancer prevention properties^11^. In addition, resveratrol acts as an important stilbene phytoalexin, which has been widely reported to possess antimicrobial ability against pathogen invasion^12–14^. These positive effects of resveratrol regarding health benefits and phytopathology have led to worldwide research on its pharmacological properties. The biosynthesis of resveratrol involves numerous enzymatic reactions, with stilbene synthases (STSs) being the most closely related and essential enzymes^15^. In the grapevine, 48 *STS* genes have been identified by sequencing the Pinot Noir PN40024 genome^16^. Among them, 33 *STSs* can be divided into three subfamilies named A to C, and B subfamily genes exhibit the greatest response to pathogen infection^17^. Stable expression of *STSs* in multiple species using transgenics might increase the content of stilbenoids and improve plant disease resistance. Hence, these *STSs* from the grapevine are resistance-related genes responsible for the accumulation of stilbene phytoalexins^18,19^.

Because of the crucial roles of *STS* genes in resveratrol biosynthesis and plant disease resistance, research in recent years has focused on exploring their upstream regulatory mechanisms. In 2013, it was reported that two transcription factors (TFs) derived from Pinot Noir grapevine, MYB14 and MYB15, activate *STS41* and *STS29* to promote stilbenoid accumulation^20^, which suggests that specific TFs in plants closely participate in the regulation of *STS* genes. Genome sequencing provides extensive data to support in-depth research on the regulatory mechanisms of TFs in plants. In grapevine, 2004 TFs have been identified by sequencing of the Pinot Noir genome^16,21^. Extensive research has shown that these TF genes are inextricably linked to the growth and development of grapevine, including cell expansion^22^, seed morphogenesis^23^, and berry ripening^24^. Moreover, numerous regulators function in defense responses to exogenous stresses, such as drought^25^, cold^26^, high salinity^27^, and pathogens^28^. Members of the WRKY^29^, NAC^28^, MYB^30^, ERF^31^, and bZIP families^32^ have been reported to play critical roles in resistance against pathogens.

R2R3-MYB TFs participate extensively in phenylpropanoid metabolism^33^; they act in functional regulation by recognizing conserved MYB binding sites (MBSs), such as the AC-box (ACCA/TAA/CT/C)^34^, MYBCORE (CAGTTA and CTGTTG)^35^, and L5-box motif (GAGTTGGTGAGA)^36^. Genome sequencing has identified these regulators in various plant species, including 138 members in *Arabidopsis*^37^, 126 in rice^38^, and 134 in grape^39^. They were initially divided into 25 subgroups in *Arabidopsis* based on the motif at the C-terminus^40^, and a later study on grapevine increased the number of subgroups to 34^39^. The numerous R2R3-MYB members may contain resistance-related TFs that are still unknown. Some resistance-related MYB proteins have been identified in *Arabidopsis*. Overexpression of *AtMYB30* leads to hypersensitive cell death in *Arabidopsis*, thereby enhancing resistance to *Pseudomonas syringae*^41^. Its homologous gene, *MdMYB30*, also has similar disease resistance properties^42^. Furthermore, *AtMYB96* enhances resistance to *P. syringae* by regulating salicylic acid (SA) biosynthesis and pathogenesis-related (*PR*) genes^43^. Knockdown of *AtMYB46* improves the resistance of *Arabidopsis* mutants to *Botrytis cinerea*^44^. In wheat, the MYB gene *TaPIMP1* was shown to promote SA-related resistance genes *PR1a* and *PR2*, thereby enhancing resistance to *Bipolaris sorokiniana*^45^. In addition to VvMYB14 and VvMYB15 in grapevine, VvMYB13 was shown to respond to infection by downy mildew and plays a positive role in stilbenoid accumulation^39^. VqMYB35 positively regulates the expression of *STS* genes by interacting with VqERF114^46^. However, considering the complexity of the MYB superfamily, more potential factors still need to be determined to fully clarify the MYB-mediated phytoalexin metabolic network.

Based on our long-term observations in vineyards, the Chinese wild-growing grapevine accession Danfeng-2 has a higher content of resveratrol and disease resistance than *V. vinifera*^4,47^. Therefore, this resistant germplasm has been used for in-depth analysis of grapevine-pathogen interactions. Recently, we determined the expression levels of 106 R2R3-MYB members in Danfeng-2 under artificial inoculation with *U. necator*, 27 of which showed greater than 4-fold upregulated expression (Supplementary Fig. S1). We then performed transcriptome analysis using Danfeng-2 berries during different developmental periods (unpublished data). Coexpression analysis based on transcriptome data was performed to identify pathogen-induced MYB TFs that regulate *STS* genes. Here, a resistance-related MYB TF, VqMYB154, from the *V. quinquangularis* accession Danfeng-2, was screened and isolated. Our research indicated that VqMYB154 is a novel regulator that improves the accumulation of stilbene phytoalexins and enhances resistance to disease in transgenic *Arabidopsis*. These results are significant for elucidating the regulatory mechanisms involved in plant-pathogen interactions and provide valuable references for the long-term goal of disease-resistant grapevine breeding.

## Results

### VqMYB154 is a resistance-related TF that participates in plant defense responses

**Fig. 1:**
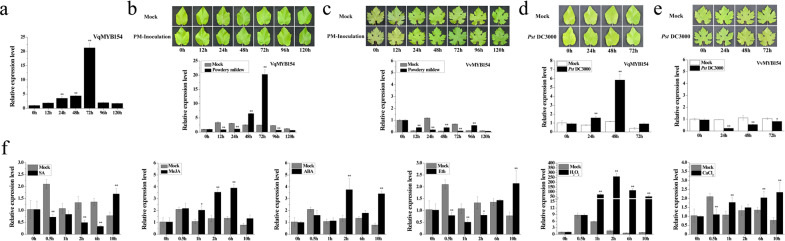
Specific expression of the transcription factor gene *VqMYB154* under artificial inoculation conditions using *U. necator* and other exogenous treatments. **a** *VqMYB154* was screened from 106 *MYB* genes in Danfeng-2 leaves infected by *U. necator* artificial inoculation under field conditions. **b**–**e** Expression of *MYB154* was determined by qRT-PCR. **b** Leaves of Danfeng-2 and (**c**) Cabernet Sauvignon were inoculated with *U. necator* and sampled at 0, 12, 24, 48, 72, 96, and 120 h. **d** Leaves of Danfeng-2 and (**e**) Cabernet Sauvignon were inoculated with *Pst* DC3000 and collected at 0, 24, 48, 72 h. *Pst* DC3000, *Pst* DC3000 infection; PM-Inoculation, *U. necator* infection; Mock, control treated with sterile water. **f** Leaves from Danfeng-2 were treated with 100 μM phytohormones, 1% (w/v) hydrogen peroxide, and 5 mM CaCl_2_. Leaves were collected at 0, 0.5, 1, 2, 6, and 10 h after spraying. Results are shown as the means (±SD) of three biological assays. Significance was determined with GraphPad Prism using one-way ANOVA with Fisher’s LSD test (*P < 0.05; **P < 0.01)

To screen out *MYB* genes involved in defense responses, we inoculated Danfeng-2 leaves with *U. necator* and performed qRT-PCR analysis. The results showed that the R2R3-type MYB gene *VqMYB154* (this study) can be induced and significantly upregulated by *U. necator* (Fig. 1a, Supplementary Fig S1). To investigate whether *MYB154* exhibits different expression patterns in disease-resistant and disease-susceptible grapes under pathogen inoculation, we inoculated the leaves of Danfeng-2 and Cabernet Sauvignon with *U. necator* and *P. syringae* (*Pst* DC3000). After artificial inoculation, the expression level of *VqMYB154* from Danfeng-2 began to increase at 48 h and peaked at 72 h, increasing 8.2-fold, compared to the mock control at the same timepoint (Fig. 1b). In contrast to *VqMYB154*, *MYB154* from Cabernet Sauvignon showed no clear expression pattern (Fig. 1c). On the other hand, after *Pst* DC3000 infection, *VqMYB154* responded to induction at 24 h and then peaked at 48 h, increasing 5.0-fold (Fig. 1d). However, *VvMYB154* exhibited a downward trend and reached its lowest abundance at 24 h (Fig. 1e). Therefore, we speculate that VqMYB154 is a resistance-related regulator in Danfeng-2.

To explore whether *MYB154* responds to exogenous phytohormones, we treated the leaves of Danfeng-2 and Cabernet Sauvignon by spraying them with phytohormones. Compared to the control at the same time, the transcript level of *VqMYB154* decreased at 0.5 h, 2 h, and 6 h but then increased 2.2-fold at 10 h after SA treatment. After MeJA treatment, the transcript level of *VqMYB154* increased at 1 h and peaked at 6 h by 2.9-fold. The transcript level of *VqMYB154* was upregulated at 2 h and peaked at 10 h by 4.4-fold after ABA treatment. Under Eth treatment, expression of *VqMYB154* decreased from 0.5 h to 2 h but then increased 2.8-fold at 10 h (Fig. 1f). On the other hand, compared to the mock control, transcript levels of *VvMYB154* were upregulated 2.4-fold at 10 h after SA treatment, 7.8-fold at 6 h after MeJA treatment, 3.3-fold at 2 h after ABA treatment, and 2.1-fold at 1 h after Eth treatment (Supplementary Fig. S3). These results demonstrate that the two *MYB154* genes respond to exogenous phytohormone induction and exhibit different expression patterns in Danfeng-2 and Cabernet Sauvignon. *VqMYB154* also responded to other exogenous signals, including H_2_O_2_ and CaCl_2_. After H_2_O_2_ treatment, expression of *VqMYB154* was significantly upregulated, reached its highest level at 2 h by 162.2-fold, and then gradually decreased. Under the induction of CaCl_2_, the expression level of *VqMYB154* was decreased at 0.5 h but increased at 1 h and 6 h and then reached its highest level at 10 h by 3.0-fold (Fig. 1f).

### Expression profiles of *VqMYB154* in ‘Danfeng-2’ grapevine

**Fig. 2:**
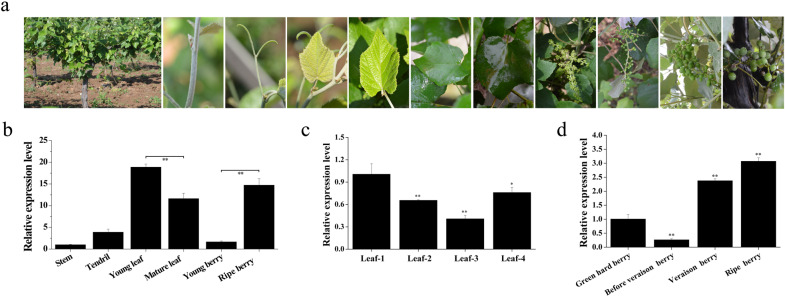
Differential expression of *VqMYB154* in various organs of Danfeng-2 under natural conditions. **a** From left to right are Danfeng-2 and its stems, tendrils, leaves during four different periods (from young to mature, leaf-1, leaf-2, leaf-3, and leaf-4), and berries of four developmental stages (green hard berry, before veraison berry, veraison berry, and ripe berry). **b** Expression analysis of *VqMYB154* in various tissues. **c** Expression analysis of *VqMYB154* in Danfeng-2 leaves in four different periods. **d** Expression analysis of *VqMYB154* in Danfeng-2 berries at four developmental stages. Results are shown as the means (±SD) of three biological assays. Significance was determined with GraphPad Prism using one-way ANOVA with Fisher’s LSD test (*P < 0.05; **P < 0.01)

After exploring the response patterns of *VqMYB154* under stress, we determined *VqMYB154* transcripts in various organs of Danfeng-2 under natural conditions (Fig. 2a). In nutritive organs, including stems, tendrils, and leaves, the expression level of *VqMYB154* in leaves was higher than that in other organs, and the expression level in young leaves was higher than that in mature leaves (Fig. 2b). The expression pattern of *VqMYB154* showed a downward trend during leaf development (Fig. 2c). In reproductive organs containing berries at the four developmental stages, expression of *VqMYB154* was higher in ripe berries than in other stages, showing an upward trend with the stage before veraison to the ripe stage (Fig. 2d). These observations indicate that young leaves and ripe berries in the Danfeng-2 grapevines are the primary sites where *VqMYB154* is expressed. Furthermore, we analyzed the expression of *VqSTS9*, *VqSTS32,* and *VqSTS42* in various organs of the Danfeng-2 grapevine. The results demonstrated that in nutritive organs, the three *VqSTS* genes showed the highest expression levels in young leaves; in reproductive organs, the three *VqSTS* genes were mainly expressed in ripe berries, which was similar to the expression pattern of *VqMYB154*. This may reflect coexpression relationships between *VqMYB154* and *VqSTS9*, *VqSTS32* and *VqSTS42* under natural conditions (Supplementary Fig. S4).

### Structure analysis and characteristics of VqMYB154

**Fig. 3:**
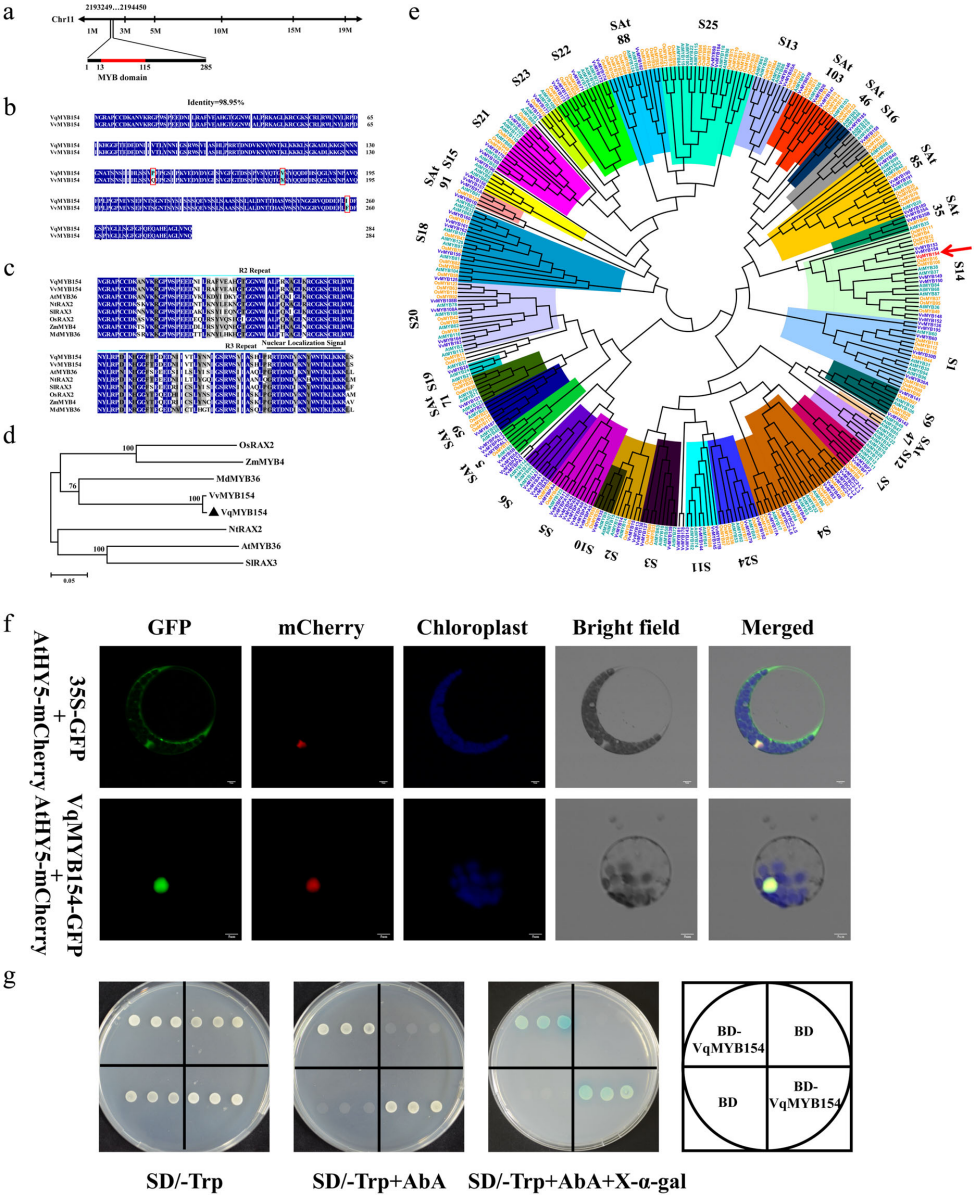
Location and structural analysis of VqMYB154 isolated from *V. quinquangularis* accession Danfeng-2. **a** Chromosomal location of *VqMYB154*. *VqMYB154* is located on chromosome 11, from position 2193249 to 2194450. The MYB domain is located at the N-terminus of VqMYB154, from aa 13 to aa 115. **b** Amino acid sequence alignments between VqMYB154 and VvMYB154. Differences are marked with a red box. **c** Multiple sequence alignments among VqMYB154 and its homologs in other species. The R2 MYB domain is indicated with a light blue line, and the R3 domain is shown with a dark blue line. The sequences are from the following: VvMYB154 (XP_002280054.2), AtMYB36 (OAO95809.1), NtRAX2 (XP_016474887.1), SlRAX3 (XP_004238554.1), OsRAX2 (XP_015637875.1), ZmMYB4 (XP_008656763.1), and MdMYB36 (XP_008337621.2). **d** Cluster analysis of VqMYB154 with its homologous genes. VqMYB154 is marked with a black triangle. **e** Cluster analysis of VqMYB154 with MYB proteins in grape, *Arabidopsis*, and rice. VqMYB154 belongs to subgroup 14 (S14) and is highly homologous to VvMYB154, VvMYB153, OsMYB12, and OsMYB72. VqMYB154 is highlighted in red and shown with a red arrow. **f** The location of VqMYB154 is in the nucleus. GFP was ligated to VqMYB154 (VqMYB154-GFP), and mCherry was ligated to the *Arabidopsis* nuclear localization marker AtHY5 (AtHY5-mCherry). The mixed plasmids were transformed together into *Arabidopsis* protoplasts. 35S-GFP was adopted as the control. The GFP and mCherry signals were detected with a confocal microscope. Bars: 5 μm. **g** Transactivation assay of VqMYB154 in yeast. Yeast strains harboring BD-VqMYB154 and the BD control were grown on SD/-Trp, SD/-Trp with AbA, and SD/-Trp with AbA and X-α-Gal medium and cultivated at 28 °C for 3 days. Only the yeast strain of BD-VqMYB154 grew and turned blue on SD/-Trp with AbA and X-α-Gal medium

To understand the function of the resistance-related gene *VqMYB154*, we first isolated *VqMYB154* and performed sequence analysis. The length of the *VqMYB154* gDNA is 1202 bp, containing two introns at positions 137–360 bp and 491–610 bp. A BLAST search in Grape Genome Browser indicated that *VqMYB154* is located on chromosome 11 (Fig. 3a). The coding sequence (CDS) of *VqMYB154* was cloned from Danfeng-2. The CDS of *VqMYB154* is 858 bp and encodes a 285 amino acid protein (Fig. 3a, b). VqMYB154 shares 98.95% amino acid identity with VvMYB154. Compared to VvMYB154, three mutations were found in the VqMYB154 protein, from glutamine to proline, methionine to valine, and phenylalanine to isoleucine (Fig. 3b). The N-terminus of VqMYB154 contains highly conserved R2 and R3 MYB domains (residues 13–115 aa) (Fig. 3a, c). An NLS motif is present at the N-terminus of VqMYB154, indicating its nuclear localization, and phylogenetic analysis of VqMYB154 with homologous proteins from multiple species demonstrated that it exhibits high homology with VvMYB154 and MdMYB36 (Fig. 3d). Cluster analysis of VqMYB154 with MYB subfamily proteins from the grapevine, *Arabidopsis*, and rice indicated that VqMYB154 is a member of subgroup 14 (S14) (Fig. 3e). *VvMYB148* in this subfamily is coexpressed with *STS* genes^48^.

To determine the subcellular site where VqMYB154 functions, we performed a subcellular localization assay, in which the GFP signal of VqMYB154 overlapped with the mCherry signal of AtHY5, indicating that VqMYB154 is a nuclear protein (Fig. 3f). In addition, yeast cells harboring BD-VqMYB154 grew normally in all cultures and showed X-α-gal activation and AbA resistance (Fig. 3g). This indicates that VqMYB154 functions as a transcriptional activator.

### The *MYB154* promoter exhibits stronger pathogen-induced activity in the resistant grapevine Danfeng-2

**Fig. 4:**
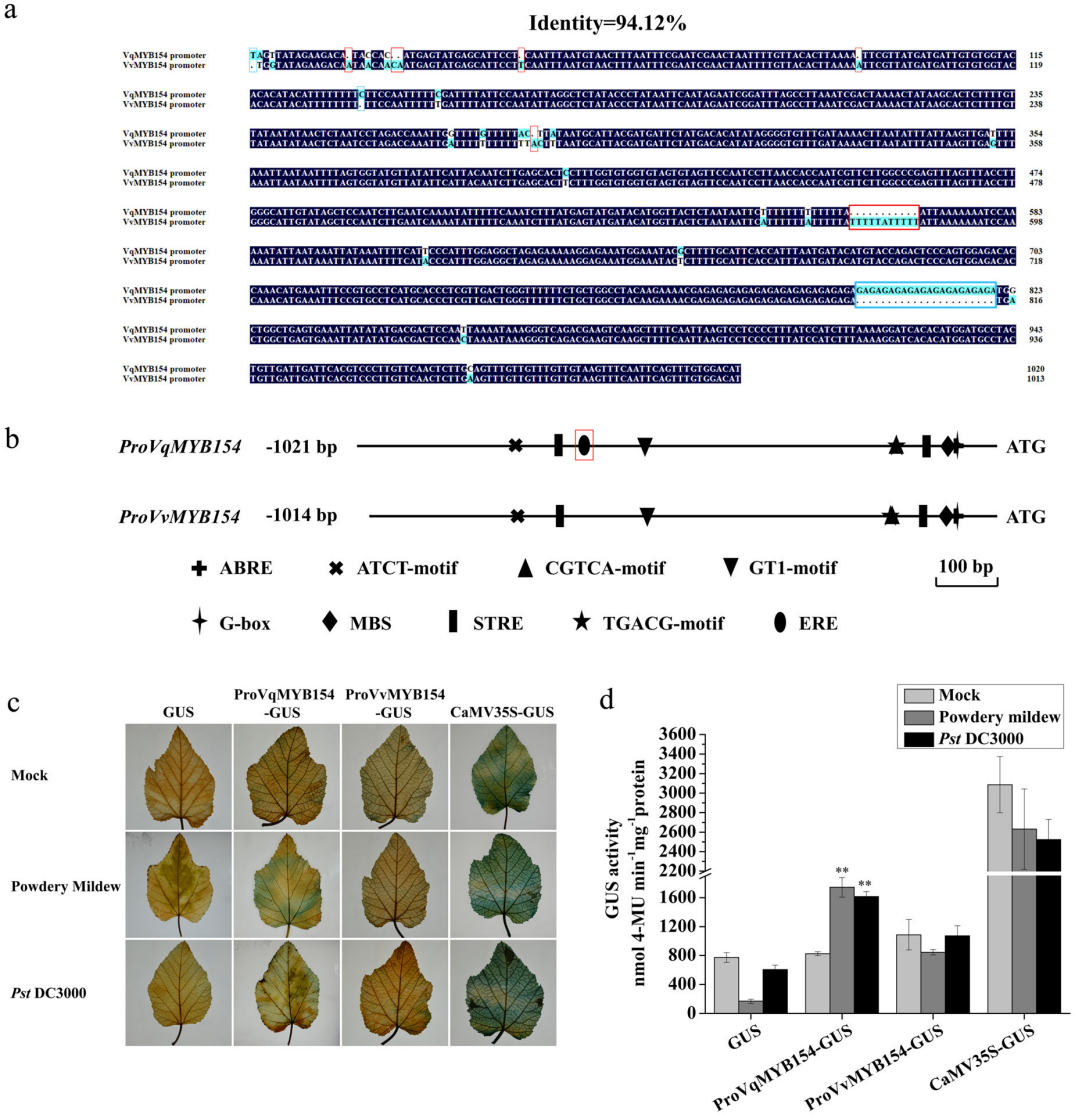
Expression activity analysis of *MYB154* promoters between Danfeng-2 and Cabernet Sauvignon induced by *U. necator*. **a** Sequence alignment between the *VqMYB154* and *VvMYB154* promoters. Deletion mutations in the *VqMYB154* promoter are marked with a red box, and the insertion mutation is marked with a blue box. **b** Cis-element analysis between the *VqMYB154* and *VvMYB154* promoters. The difference in cis-element in the *VqMYB154* promoter is marked with a red box. **c** Histochemical staining of GUS activity mediated by *VqMYB154* and *VvMYB154* promoters under *U. necator* and *Pst* DC3000 inoculation in grapevine leaves. Transiently transformed samples were cultivated for 48 h in a phytotron before inoculation with pathogens for 24 h. **d** Determination of GUS expression mediated by transient expression of *VqMYB154* and *VvMYB154* promoters under *U. necator* and *Pst* DC3000 inoculation in grapevine leaves. Results are shown as the means (±SD) of three biological assays. Significance was determined with GraphPad Prism using one-way ANOVA with Fisher’s LSD test (*P < 0.05; **P < 0.01)

Previous results have suggested that *MYB154* from disease-resistant and disease-susceptible grapevines shows different pathogen-induced response patterns. To further explore the basis for these differences in expression, we separately cloned and obtained the promoters of *MYB154* in Danfeng-2 and Cabernet Sauvignon. Compared with the 1014 bp promoter of *VvMYB154*, we detected six deletion mutations and three insertion mutations in the 1021 bp promoter of *VqMYB154* (Fig. 4a). We further discovered numerous motifs in the *VqMYB154* promoter, including an MBS element, ABRE element (ABA-responsive), CGTCA motif, TGACG motif (MeJA-responsive), ERE element (ethylene-responsive), and STRE element (stress-responsive). This suggests a potential role for *VqMYB154* in multiple stress responses (Fig. 4b).

To investigate the effect of promoter differences on pathogen-induced response activity, we transiently transformed the two promoters into grape leaves and evaluated GUS activity in leaves inoculated with *U. necator* and *P. syringae* (*Pst* DC3000). Compared to the mock control, GUS activity driven by the *VqMYB154* promoter was significantly enhanced after inoculation with *U. necator* and *Pst* DC3000, whereas GUS activity of the *VvMYB154* promoter showed no significant change after pathogen infection (Fig. 4c, d). These results indicate that *VqMYB154* possesses a pathogen-inducible promoter that responds to both fungal and bacterial pathogens, and the differential expression of *MYB154* genes in pathogen-inoculated leaves is closely associated with the activity of their promoters.

### *VqMYB154* enhances the expression of *STS* genes and stilbenoid synthesis

**Fig. 5:**
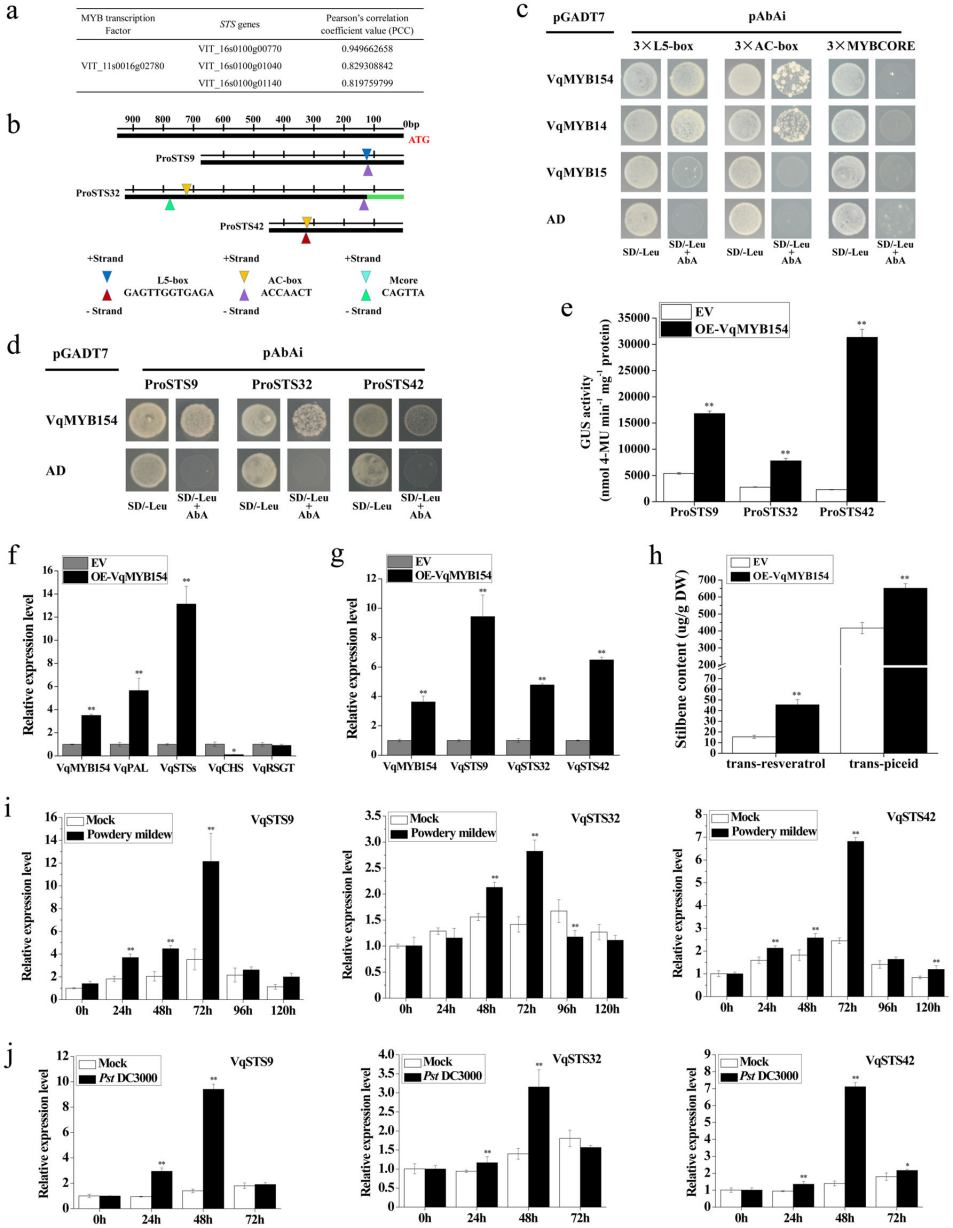
Expression of three *VqSTS* genes and the biosynthesis of stilbene phytoalexin regulated by *VqMYB154*. **a** Coexpression of *VqMYB154* and *STS* genes. **b** Graphical analysis of *VqSTS9*, *VqSTS32*, and *VqSTS42*. L5-box (GAGTTGGTGAGA), AC-box (ACCAACT), and MYBCORE element (CAGTTA) are MYB binding cis-elements. **c** A yeast one-hybrid experiment indicated that VqMYB154 has the ability to bind to L5 box and AC box elements. The CDS of VqMYB154 was integrated into pGADT7 to form AD-VqMYB154. AD-VqMYB14 and AD-VqMYB15 fusion vectors were used for the comparison of binding ability with VqMYB154. The pGADT7 vector was adopted for controls. The yeast strains harboring three tandem repeats of GAGTTGGTGAGA, ACCAACT, and CAGTTA were used as bait. Transformants were grown on medium with SD/ − Leu and AbA. **d** A yeast one-hybrid experiment indicating that VqMYB154 interacts with *STS* promoters. Y1H strains harboring the promoters of *VqSTS9*, *VqSTS32*, and *VqSTS42* were used as bait. The pGADT7 vector was adopted for controls. **e** VqMYB154 activates *VqSTS9*, *VqSTS32*, and *VqSTS42* promoters. The vectors P_VqSTS9_-GUS, P_VqSTS32_-GUS, and P_VqSTS42_-GUS were cotransformed with VqMYB154 into tobacco leaves. Samples were collected at 72 h after transformation. EV, the empty vector pCAMBIA1391, was used as the negative control. **f** Transient overexpression of *VqMYB154* promotes the expression of genes related to the stilbene biosynthesis pathway. *PAL*, *Phenylalanine ammonia lyase* gene. *VqSTSs* represent the total *STS* genes in the grapevine, as amplified based on the homologous sequences of *STS* genes. *RSGT*, *resveratrol glycosyl transferase* gene. The *chalcone synthase* gene (*CHS)*, which functions in the flavonoid pathway, was utilized as the control gene. Overexpression of *VqMYB154* was performed in *Agrobacterium*-transformed leaves of Danfeng-2. EV, empty vector was adopted as the control. **g** Transient overexpression of *VqMYB154* promotes expression of *VqSTS9*, *VqSTS32*, and *VqSTS42*. EV, the empty vector, was used as the control. **h** Overexpression of *VqMYB154* promotes stilbenoid biosynthesis. Accumulation of trans-piceid and trans-resveratrol was determined by HPLC. **i**, **j** Expression of *VqSTS9*, *VqSTS32,* and *VqSTS42* was determined by qRT-PCR. **i** Leaves of Danfeng-2 were inoculated with *U. necator* and sampled at 0, 24, 48, 72, 96, and 120 h. **j** Leaves of Danfeng-2 were inoculated with *Pst* DC3000 and collected at 0, 24, 48, 72 h. PM-Inoculation, *U. necator* infection; *Pst* DC3000, *Pst* DC3000 infection; Mock, control treated with sterile water. Results are shown as the means (±SD) of three biological assays. Significance was determined with GraphPad Prism using one-way ANOVA with Fisher’s LSD test (*P < 0.05; **P < 0.01)

To identify the downstream target gene of VqMYB154, we conducted gene correlation analysis using Danfeng-2 transcriptome data. The Pearson correlation coefficient (PCC) was adopted as the key index for analyzing correlations^49^. In particular, we noticed that *VqMYB154* (VIT_11s0016g02780) was coexpressed with *STS9* (VIT_16s0100g00770), *STS32* (VIT_16s0100g01040), and *STS42* (VIT_16s0100g01140) according to high PCC indexes of 0.94, 0.82, and 0.81 (Fig. 5a), respectively. Thus, VqMYB154 may act as a regulator of the stilbene pathway. To test this hypothesis, we amplified and obtained these three promoters using Danfeng-2 genomic DNA. By analyzing the sequences, we found that the *VqSTS9* promoter contains an MYB binding motif L5-box and AC-box and that the *VqSTS32* promoter possesses two AC-boxes and an MYBCORE element. In addition, the *VqSTS42* promoter contains an AC box and L5 box element (Fig. 5b). Therefore, we deduced that these elements are binding sites for VqMYB154. We then used a yeast one-hybrid (Y1H) assay to examine the ability of VqMYB154 to bind to these motifs. VqMYB14 and VqMYB15 from Danfeng-2, which have been proven to promote the expression of *STS* genes^50^, were used for comparison. The results indicated that VqMYB154 is able to bind to the L5-box and AC-box motifs but not the MYBCORE motif, which is consistent with the results for VqMYB14 but not VqMYB15 (Fig. 5c). A further Y1H assay demonstrated that VqMYB154 interacts with the promoters of *VqSTS9*, *VqSTS32*, and *VqSTS42* (Fig. 5d). To determine whether the impact of these interactions is positive or negative, we next performed a GUS activity determination assay in tobacco. The results demonstrate that transient overexpression of VqMYB154 activated the promoters of these *VqSTS* genes in vivo (Fig. 5e).

After determining the role of VqMYB154 in activating *STS* promoters, we investigated the effect of VqMYB154 on several node genes in the phenylalanine pathway and assessed the transcriptional levels of genes, including *phenylalanine ammonia lyase* (*PAL*), *chalcone synthase* (*CHS*), total *VqSTSs* (total expression level of *STS* genes), and *resveratrol glycosyl transferase* (*RSGT*), by performing transient overexpression assays in Danfeng-2 leaves. The results showed that overexpression of *VqMYB154* led to activation of *PAL* and total *VqSTSs* but that the *CHS* gene was inhibited. Expression of *RSGT* was unaffected by *VqMYB154* (Fig. 5f). Moreover, as expected, *VqSTS9*, *VqSTS32*, and *VqSTS42* were activated in *VqMYB154*-overexpressing leaves (Fig. 5g). The HPLC assay indicated that the contents of trans*-*resveratrol and trans*-*piceid increased 2.9-fold and 1.6-fold, respectively, after VqMYB154 overexpression (Fig. 5h). Together, VqMYB154 can promote phenylalanine metabolism and the downstream STS pathway while inhibiting the flavonoid pathway.

We further analyzed the expression patterns of *VqSTS* genes in Danfeng-2 leaves under pathogen inoculation and found that *VqSTS9*, *VqSTS32*, and *VqSTS42* can respond to induction by *U. necator*. Compared to the mock control, transcriptional levels of *VqSTS9* and *VqSTS42* were significantly upregulated at 24 h and peaked at 72 h by 3.4-fold and 2.8-fold, respectively. The transcriptional level of *VqSTS32* was increased at 48 h and peaked at 72 h by 2.0-fold (Fig. 5i). Under inoculation with *Pst* DC3000, expression of the three *STS* genes was enhanced at 24 h and peaked at 48 h by 6.7-fold, 2.2-fold and 5.1-fold, respectively. Furthermore, three *VqSTS* genes shared the same upregulated periods with *VqMYB154* under the induction of *U. necator* (48 h and 72 h) and *Pst* DC3000 (24 h and 48 h), which indicates that *VqMYB154* is coexpressed with *VqSTS9*, *VqSTS32*, and *VqSTS42* under pathogen-induced conditions (Fig. 5j).

### *VqMYB154* is a positive regulator of resistance to *Pseudomonas syringae* in transgenic *Arabidopsis*

**Fig. 6:**
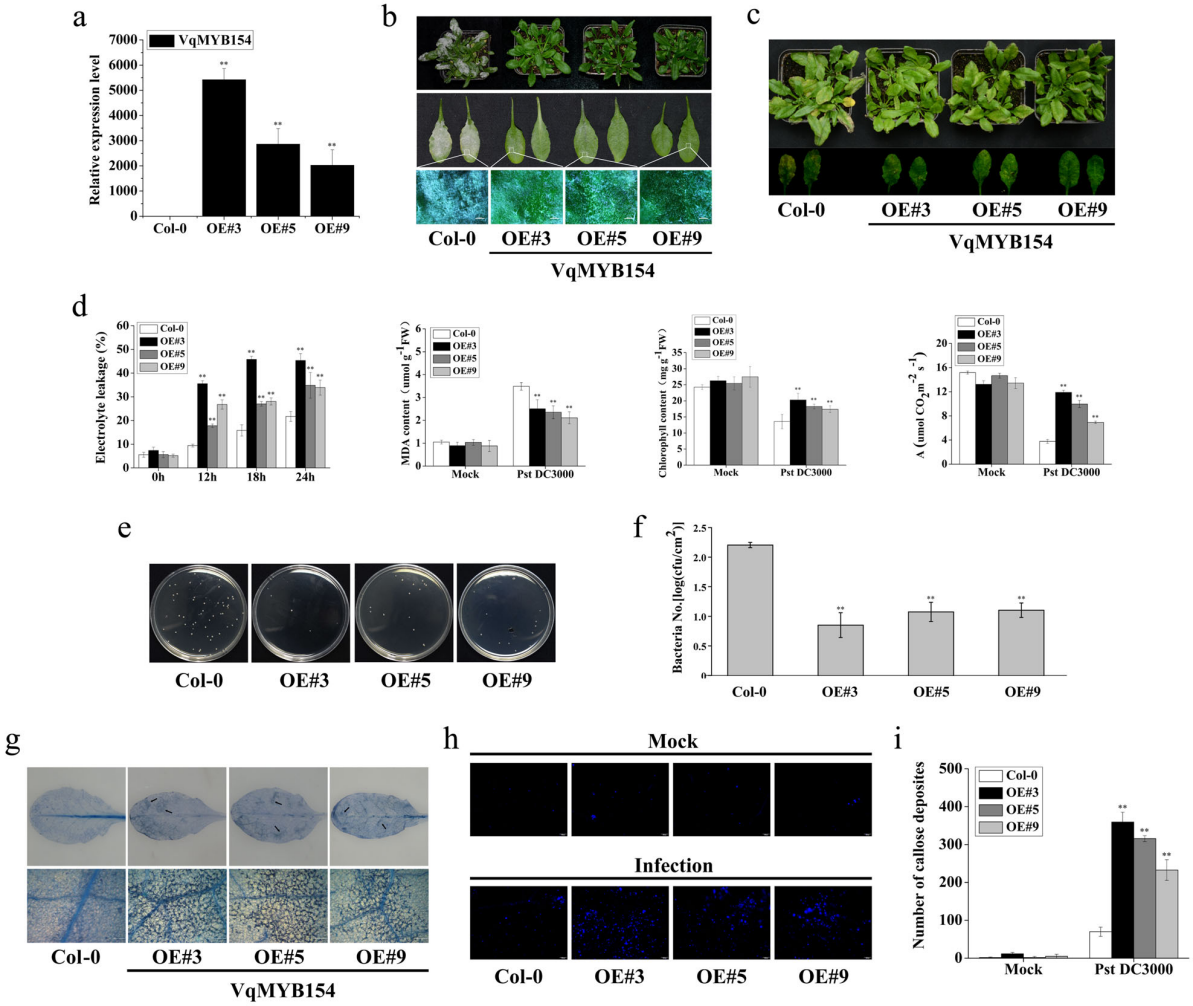
Overexpression analysis of *VqMYB154* in *Arabidopsis* under artificial inoculation with pathogens. **a** *VqMYB154* was overexpressed in OE#3, OE#5, and OE#9 *Arabidopsis* transgenic lines. **b** Phenotype of leaves from wild-type (Col-0) and transgenic lines (OE#3, OE#5, and OE#9) after inoculation with *G*. *cichoracearum* UCSC1 for 168 h. The figures from top to bottom are as follows: overall observation of *Arabidopsis* plants, close observation of leaf phenotype, and microscopic observation (Bars = 500 μm) of hyphae on the leaf surface. **c** Phenotype of leaves from wild-type (Col-0) and transgenic lines (OE#3, OE#5, and OE#9) after infection with *Pst* DC3000 (2 × 10^6^ cfu ml^−1^ MgCl_2_ suspension) for 72 h. **d** Physiological indexes were measured in leaves of wild-type (Col-0) and transgenic lines (OE#3, OE#5, and OE#9) after infection with *Pst* DC3000 (8 × 10^6^ cfu ml^−1^ MgCl_2_ suspension). The indexes from left to right are electrolyte leakage, malondialdehyde (MDA) content, total chlorophyll content, and assimilation rate. Electrolyte leakage was determined at 0 h, 12 h, 18 h, and 24 h, and other indexes were detected after inoculation for 72 h. The assimilation rate (A) represents the net photosynthetic rate. **e**, **f** The measurement of bacterial colonies in *Arabidopsis* leaf samples at 72 h after inoculation with *Pst* DC3000 (1 × 10^3^ cfu ml^−1^ MgCl_2_ suspension). **e** Bacterial colonies from leaf samples were generated in Petri dishes, and (**f**) quantities were counted. **g** Trypan blue staining was performed to detect cell death after infection with *Pst* DC3000 (1 × 10^3^ cfu ml^−1^ MgCl_2_ suspension) for 72 h. Bars: 200 μm. **h** Aniline blue staining was performed to detect callose deposition in the leaves 24 h after 1 × 10^3^ cfu ml^−1^ *Pst* DC3000 inoculation. Bars: 100 μm. **i** Count of callose deposition in the microscopic field using ImageJ software. Results are shown as the means (±SD) of three biological assays. Significance was determined with GraphPad Prism using one-way ANOVA with Fisher’s LSD test (*P < 0.05; **P < 0.01)

*VqMYB154* and its promoter respond to induction by pathogens (Fig. 1, Fig. 4). To further explore its function in the defense response, we generated *VqMYB154-*overexpressing *Arabidopsis* lines. Wild-type (Col-0) plants and *VqMYB154* transgenic lines (OE#3, OE#5, and OE#9) were used for disease assays (Fig. 6a). We first inoculated transgenic lines and Col-0 plants with *Golovinomyces cichoracearum*. At 168 h postinoculation, the leaves of three independent transgenic lines showed fewer fungal spores and hyphae than did Col-0 plants, indicating the transgenic lines to be more resistant to *G*. *cichoracearum* (Fig. 6b). In addition, *VqMYB154*-overexpressing transgenic lines showed stronger resistance to *Pst* DC3000. At 72 h postinoculation, the transgenic lines exhibited less severe chlorosis symptoms than did Col-0 plants (Fig. 6c). To further evaluate stress tolerance under *Pst* DC3000 inoculation, resistance-related physiological indexes were measured. Notably, electrolyte leakage from transgenic lines was significantly increased, higher than that of Col-0 plants, within 24 h postinoculation, which may indicate that a more intense hypersensitive reaction occurred in the transgenic lines. Furthermore, consistent with the phenotype of the inoculated plants, the transgenic lines had a lower malonaldehyde content and higher chlorophyll content and net photosynthetic rate than wild-type plants (Fig. 6d). We hypothesize that these phenotypic differences may be linked to the growth status of the pathogens in vivo. Therefore, we measured the abundance of bacteria in leaves at 72 h after *Pst* DC3000 inoculation and found them to be significantly lower than those of Col-0 plants (Fig. 6e, f). A trypan blue assay was performed to visualize areas of cell death in transgenic lines and Col-0 plants, showing more intense cell death at 72 h after *Pst* DC3000 inoculation (Fig. 6g). By staining leaf samples with aniline blue, we observed more intensive callose deposition in the transgenic lines at 72 h after *Pst* DC3000 inoculation than in wild-type plants. The amount of callose in the transgenic lines was also significantly increased (Fig. 6h, i). These results indicate that *VqMYB154* can enhance disease resistance against *Pst* DC3000.

### *VqMYB154* stimulates the production of reactive oxygen species (ROS) in vivo and enhances resistance to *P. syringae* via the SA pathway

**Fig. 7:**
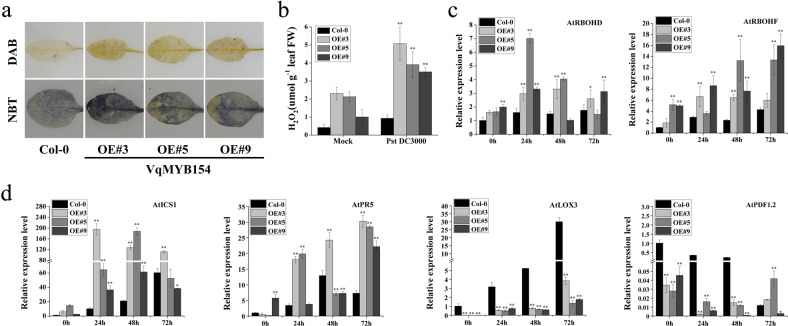
Overexpression of *VqMYB154* in *Arabidopsis* stimulates ROS accumulation in vivo and enhances the expression of defense-related genes. **a** DAB staining and NBT staining were utilized to detect ROS (H_2_O_2_ and O^2−^) production after infection with *Pst* DC3000 (1 × 10^3^ cfu ml^−1^ MgCl_2_ suspension) for 72 h. From top to bottom: DAB staining and NBT staining. **b** Measurement of the H_2_O_2_ content in leaves after infection of *Pst* DC3000 (1 × 10^3^ cfu ml^−1^ MgCl_2_ suspension) for 72 h. **c** qRT-PCR analysis at the transcriptional level of the NADPH oxidase genes *AtRBOHD* and *AtRBOHF* in *Arabidopsis* transgenic lines and wild-type at 0, 24, 48, and 72 h after 1 × 10^3^ cfu ml^−1^ *Pst* DC3000 infection. **d** qRT-PCR analysis of expression levels of defense-related genes *AtICS1*, *AtPR5*, *AtLOX3*, and *AtPDF1.2* in transgenic lines and wild-type at 0, 24, 48, and 72 h under 1 × 10^3^ cfu ml^−1^ *Pst* DC3000 inoculation. Results are shown as the means (±SD) of three biological assays. Significance was determined with GraphPad Prism using one-way ANOVA with Fisher’s LSD test (*P < 0.05; **P < 0.01)

To investigate whether ROS participate in the defense response, we performed DAB and NBT staining to visualize the contents of H_2_O_2_ and O^2−^. We observed that more ROS accumulated in *VqMYB154-*overexpressing transgenic lines than in wild-type plants at 72 h after *Pst* DC3000 inoculation (Fig. 7a). We also quantified endogenous H_2_O_2_ content. As expected, the H_2_O_2_ content in vivo was higher in transgenic plants, which is consistent with the above results (Fig. 7b). The further qRT-PCR analysis demonstrated that transcriptional levels of the *NADPH* oxidase genes *AtRBOHD* and *AtRBOHF*, which are defense-related genes involved in ROS production^51,52^, were upregulated in transgenic plants and higher than those in Col-0 plants at 24 h and 48 h postinoculation (Fig. 7c).

Our results indicate that *VqMYB154* can respond to the defense-related phytohormones SA and MeJA (Fig. 1f). To explore whether VqMYB154 can activate resistance signaling pathways that involve SA or JA, we analyzed SA-independent and JA-independent defense genes in transgenic lines and Col-0 plants under *Pst* DC3000 inoculation. *AtICS1* participates in SA biosynthesis for plant defense responses^53^, and *AtPR5* is an important SA-independent resistance-related gene^54^. After pathogen inoculation, the expression level of *AtICS1* in transgenic lines increased at 24 h and 48 h compared to wild-type. Expression of *AtPR5* in transgenic lines was more intense than that in Col-0 plants at 72 h postinoculation (Fig. 7d). However, transcript levels of *AtLOX3* and *AtPDF1*.2, which are JA-independent defense genes^55,56^, were significantly lower than those of wild-type (Fig. 7d). These results demonstrate that overexpression of *VqMYB154* stimulates ROS accumulation and enhances resistance to *P. syringae* via the SA signaling pathway.

## Discussion

Grape diseases can cause yield loss and even depression in the grape industry. Disease-resistant grapevines possess unclarified grapevine-pathogen interaction mechanisms and elucidating transcription networks regulating plant resistance is vital for viticulture and vine breeding. In this study, we cloned and identified a novel resistance-related R2R3-MYB gene, *VqMYB154*, from the disease-resistant grapevine Danfeng-2. This research focused on elucidating its role in phytoalexin biosynthesis and defense responses. We found that pathogens are direct factors resulting in the activation of *VqMYB154* and its promoter and further determined its involvement in the biosynthesis of stilbene phytoalexin by identifying its target *STS* genes. We then evaluated its resistance-related function using assays in *VqMYB154*-overexpressing *Arabidopsis* mutants. In summary, our results reveal that VqMYB154 positively contributes to plant defense responses.

### VqMYB154 is a resistance-related transcription factor that participates in the plant defense response

The Chinese wild-growing *V*. *quinquangularis* accession Danfeng-2 shows excellent resistance to exogenous pathogens, including *U. necator*^4^. TFs involved in plant defense mechanisms can respond to pathogen induction^57^. In our study, *VqMYB154*, an MYB TF isolated from resistant Danfeng-2, significantly responded to *U. necator* and *Pst* DC3000 (Fig. 1a–c). However, its homolog in susceptible Cabernet Sauvignon is insensitive to pathogen invasion. Thus, *VqMYB154* differs from *VvMYB154*, and there is a specific TF gene involved in the resistance mechanism of Danfeng-2. Furthermore, we found that various mutations, including insertions and deletions, exist in the *VqMYB154* promoter (Fig. 4). Homologs may behave differently, even within the same species. For example, the ubiquitin ligase gene *RH2* enhances resistance to powdery mildew in Chinese wild-growing grapevine Baihe-35-1 but not in susceptible cultivars because of mutations in the promoter sequence and differences in promoter elements^58^. OsMYBS1 binds to the promoter of C_2_H_2_-type TF Bsr-d1 in disease-resistant rice Digu to improve resistance to rice blast, whereas, in susceptible rice, such binding does not occur because of a single-nucleotide variation ‘G’ to ‘A’ in the promoter^59^. Therefore, we propose that the mutations between the two *MYB154* genotypes are directly responsible for the different promoter activities. This is also the reason why the expression of *VqMYB154* is more prominent in the disease response.

Previous studies have reported that *P. syringae* can trigger incompatible interactions and innate defense responses in grapevines^60,61^. It is speculated that *Pst* DC3000 is a nonadapted pathogen for grapevine. Furthermore, interactions of *Pst* DC3000 with grapevine induce expression of resistance-related TFs, including ERF112, ERF114, and ERF072^31^. Therefore, we combined *U. necator* and *Pst* DC3000 together to investigate defense-related gene expression in the grapevine. The results showed that *VqMYB154* was induced and exhibited upregulated expression under artificial inoculation with *Pst* DC3000. This may reflect that VqMYB154 also plays a role in incompatible grapevine-pathogen interactions, which deserves further research.

### VqMYB154 activates the STS pathway and enhances stilbenoid accumulation

Resveratrol is an attention-attracting metabolite because of its antimicrobial activity in plants and positive pharmacological effects on human health^11,62^. It is particularly abundant in grape berries and gradually accumulates from veraison to the ripe period^63,64^. In recent years, several MYB proteins have been identified as resveratrol regulators^20,30^. *VviMYB14*, *VviMYB15*, and *VviMYB13*, which regulate the STS pathway, belong to MYB subgroup 2. Other identified MYB genes that are coexpressed with *STS* genes include *VviMYB139* (subgroup 3), *VviMYB148* (subgroup 14), and *VviMYB108A* (subgroup 20)^48^, and *VqMYB154* in this study belong to the same subfamily as *VviMYB148* (Fig. 3e). According to the grapevine expression atlas from *V*. *vinifera* L. cv. Corvina, *VviMYB14,* and *VviMYB15* are expressed during the developmental periods of berries, leaves, stems, and tendrils, while *VviMYB154* is only expressed in young leaves and tendrils^65^. In contrast to its homolog in Corvina, *VqMYB154* is expressed in multiple organs, and its mRNA accumulates significantly in veraison and ripe berries (Fig. 2). Expression levels of *VqSTS9*, *VqSTS32,* and *VqSTS42* in ripe berries were higher than those in young berries (Supplementary Fig. S5). These results indicate that *VqMYB154* is similar to *VviMYB14* and *VviMYB15* in the expression distribution in grapevine organs and provides evidence for the correlation between VqMYB154 and the STS pathway under natural conditions.

Specific binding elements act as a “bridge” between transcription factors and their target genes. In grapevines, MYB14 recognizes and binds to the L5-box element (GAGTTGGTGAGA) in the *STS* promoter to regulate stilbene accumulation^36^. In addition, the AC box (ACCA/TAA/CT/C) and MYBCORE (CAGTTA and CTGTTG) serve as cis-acting sites for MYB proteins^34,35^. In our study, the promoters of *VqSTS9* and *VqSTS42* were found to contain both an L5-box GAGTTGGTGAGA and AC-box ACCAACT. The promoter of *VqSTS32* harbors the AC-box ACCAACT and MYBCORE CAGTTA (Fig. 5b). The existence of these specific sites indicates that *STS* genes may be regulated by MYB TFs. As a control TF for comparing binding properties, VqMYB14 can bind to the L5-box element in Danfeng-2, which is consistent with a previous study on grapevine^36^. Moreover, we confirmed that the AC box acts as a novel binding element for VqMYB14. Furthermore, VqMYB15 can weakly interact with the L5 box but cannot bind to the other two elements, indicating that it may bind to other cis-elements with better affinity in *STS* promoters. Interestingly, VqMYB154 shares the same binding preferences as VqMYB14. This suggests that VqMYB154 performs transcriptional regulatory functions in a manner similar to that of VqMYB14.

Because of their common evolutionary origin, STS and CHS compete for the same substrates, such as *p*-coumaroyl-CoA^15^. As an upstream enzyme, PAL participates in *p*-coumaroyl-CoA biosynthesis. Therefore, activating the *PAL* gene can create more substrates to be used for resveratrol biosynthesis^20^. Our results show that VqMYB154 not only enhances expression of the three *STS* genes but also upregulates *VqPAL* expression and downregulates that of *VqCHS* (Fig. 5f, g). More substrates weakened CHS competitiveness, and higher STS activity promotes the accumulation of stilbenes. As expected, we detected higher levels of trans-piceid and trans-resveratrol in *VqMYB154*-overexpressing grape leaves than in the control (Fig. 5h). Taken together, VqMYB154 is a transcriptional activator of resveratrol accumulation.

### *VqMYB154* contributes to resistance against *P. syringae* in transgenic *Arabidopsis*

Plants have developed a series of emergency mechanisms to resist pathogens. Programmed cell death (PCD) leads to acute necrosis in infected cells, interrupting pathogen spread^66^. To date, only a few MYB TFs have been shown to directly regulate pathogen-triggered PCD^67,68^. Our results showed that heterologous expression of *VqMYB154* in *Arabidopsis* leads to more intense PCD upon *Pst* DC3000 infection (Fig. 6f). Moreover, we noted that regional cell necrosis is accompanied by more reactive oxygen species (ROS) generation (Fig. 7a–c). ROS, such as H_2_O_2_, can resist pathogen invasion by inducing resistance-related gene expression and further PCD^69^. In fact, it is commonly accepted that ROS and SA signals function synergistically in systemic acquired resistance^70^. Consistent with this viewpoint, we also detected upregulated expression of SA-dependent *AtICS1* and *AtPR5* following *Pst* DC3000 inoculation, indicating activation of the SA signaling pathway (Fig. 7d). Interestingly, *VqMYB154* responded intensively to H_2_O_2_ induction in grapevines (Fig. 1f), and the endogenous H_2_O_2_ generated by pathogenic stimulation might further induce expression of *VqMYB154*, possibly constituting a positive feedback loop mediated by ROS. Furthermore, we observed that callose deposition was denser in transgenic lines upon *Pst* DC3000 inoculation (Fig. 6g). Overall, enhanced callose accumulation can more effectively protect cells against pathogen penetration^71^.

**Fig. 8:**
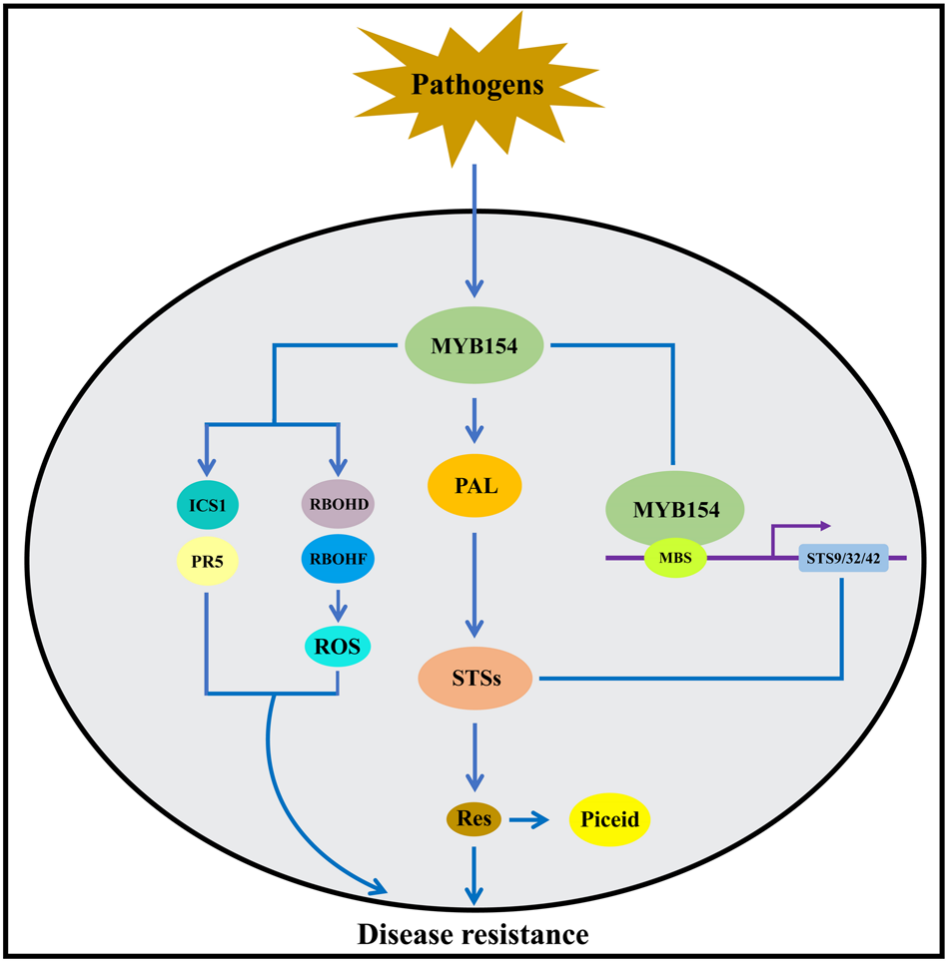
Proposed model of how VqMYB154 regulates stilbene phytoalexin biosynthesis and disease resistance. Pathogens induce expression of *VqMYB154*. VqMYB154 can act in two pathways. First, VqMYB154 activates MBS elements in *STS* promoters to induce gene expression. Furthermore, *VqMYB154* promotes the expression of the upstream *PAL* gene to provide more substrate for stilbene synthases, which biosynthesize more stilbenoids. The accumulation of stilbene phytoalexins enhances plant immunity. In addition, VqMYB154 stimulates ROS accumulation and upregulates the SA signaling-related genes *AtICS1* and *AtPR5*. These defense-related genes and ROS accumulation also contribute to disease resistance

In summary, our research reveals that multiple resistance-related molecular mechanisms comprise the *VqMYB154*-mediated plant defense response. *VqMYB154* is activated by pathogens and promotes stilbenoid biosynthesis by activating the promoters of *STS* genes. Moreover, *VqMYB154* promotes the expression of *PAL* and inhibits that of *CHS*, further activating the resveratrol pathway. Thus, the accumulation of resveratrol contributes to plant disease resistance. In addition, VqMYB154 activates downstream resistance-related genes and ROS production, which leads to enhanced disease resistance. The ROS accumulated might further induce continuous expression of *VqMYB154* and form a positive feedback pathway (Fig. 8). Overall, our research provides new insight into the mechanism of transcriptional regulation in phytoalexin metabolism and the plant defense response, offering valuable evidence for utilizing Danfeng-2 as an important resource for grapevine breeding.

## Materials and methods

### Plant materials

Organs from Danfeng-2 and Cabernet Sauvignon were acquired from the Grape Germplasm Resources database at Northwest A&F University, Yangling, Shaanxi, China (34°20′N, 108°24′E). Grape berries were acquired during four developmental periods: green hard, before veraison, veraison, and ripe. The collection times were 25, 40, 50, and 80 days after flowering. The leaf collection periods were the same as those of berries. Based on the condition of the leaves, they were denoted Leaf-1, Leaf-2, Leaf-3, and Leaf-4. Tobacco (*Nicotiana benthamiana*) was cultivated in a climate chamber at 25 °C. *Arabidopsis* (Columbia ecotype) was cultivated in a plant incubator at 22 °C.

### Gene isolation and bioinformatics analysis

cDNA from Danfeng-2 berries was used for gene amplification. With reference to the sequence of homologous genes in Pinot Noir, the *VqMYB154* coding sequence (CDS) (VIT_11s0016g02780) was cloned using the primers VqMYB154-F/VqMYB154-R (Supplementary Table S1). Sequence alignment was analyzed using DNAMAN (Lynnon Biosoft, San Ramon, CA, USA). Cluster analysis was performed using MEGA 10.1.8 (Pennsylvania State University, University Park, USA) and FigTree (Andrew Rambaut, Institute of Evolutionary Biology, UK). Chromosomal localization was analyzed using Grape Genome Browser (http://www.genoscope.cns.fr/externe/GenomeBrowser/Vitis/). Conserved protein domains were determined on the website SMART (http://smart.embl-heidelberg.de/). The sequence of nuclear localization was analyzed using the SeqNLS website (http://mleg.cse.sc.edu/seqNLS/).

### Artificial inoculation of *U. necator* and *P. syringae* under field conditions

*U. necator* was collected from the surface of susceptible grape leaves. Infection of Danfeng-2 and Cabernet Sauvignon leaves with *U. necator* was based on a previously described method^4^. Petioles of inoculated leaves were wrapped in moist, medical absorbent cotton and placed in flat trays with wet filter paper padded inside. The infected leaves were sampled at 0, 12, 24, 48, 72, 96, and 120 h, were wrapped in marked tin foil, immediately stored in liquid nitrogen, and placed in a cryogenic refrigerator for experiments. *P. syringae* (*Pst* DC3000) was cultivated in liquid medium^72^ with 25 mg/L rifampicin added in an orbital shaker (28 °C; 2 days). Healthy grape leaves were infiltrated with a suspension of *Pst* DC3000, and the inoculated leaves were collected at 0, 24, 48, and 72 h.

### Treatments using phytohormone and abiotic stress

Leaves of Danfeng-2 and Cabernet Sauvignon were used for phytohormone treatment. SA, abscisic acid (ABA), methyl jasmonate (MeJA), and ethylene (Eth) were prepared with absolute ethanol and then diluted with double distilled water to 100 μM. Hydrogen peroxide (H_2_O_2_; 1% [w/v]) and 5 mM CaCl_2_ were used for the treatment of Danfeng-2 leaves. The leaves of the control group were treated with double-distilled water. Treated leaves were collected after 0, 0.5, 1, 2, 6, and 10 h.

### Subcellular localization for VqMYB154 analysis

The *VqMYB154* CDS (stop codon excluded) was ligated to the pCAMBIA2300 vector^73^, and the 35S-VqMYB154-GFP plasmid generated was used in this assay. The recombinant vector 35S-AtHY5-mCherry acted as a marker for the nucleus^74^. The pCAMBIA2300 vector was used as the control. The plasmids (35S-AtHY5-mCherry+35S-VqMYB154-GFP and 35S-AtHY5-mCherry+35S-GFP) were transfected into *Arabidopsis* leaf protoplasts and cultivated for 22 h based on a previous protocol^75^. GFP and mCherry signals were detected using a laser scanning confocal microscope (Olympus FV1000MPE, Tokyo, Japan). The color of the chloroplast signal in the figure is shown in blue to distinguish it from mCherry fluorescence.

### Yeast transactivation assay of VqMYB154

The CDS of *VqMYB154* was ligated to the pGBKT7 vector (Clontech, Mountain View, CA, USA). The BD-VqMYB154 plasmid generated was transferred into the Y2HGold strain (Clontech). The pGBKT7 vector served as the control. The transformed strains were cultured on SD/-Trp medium at 28–30 °C for 3 days, and transformants were grown on Petri dishes and cultivated at 28–30 °C for 3 days before observation. Three types of media were used: SD/-Trp, SD/-Trp with aureobasidin A (AbA), and SD/-Trp with AbA or X-α-Gal.

### Yeast one-hybrid for screening promoter assays

Matchmaker™ Gold Yeast One-Hybrid System (Clontech, Palo Alto, USA) was adopted for experimental validation. The *STS* promoters of *VqSTS9*, *VqSTS32*, and *VqSTS42* were integrated into the pAbAi vector to form pAbAi-ProVqSTS9, pAbAi-ProVqSTS32, and pAbAi-ProVqSTS42. Three tandem copy sequences of ACCAACT (AC-box), GAGTTGGTGAGA (L5-box), and CAGTTA (MYBCORE) were also integrated into the pAbAi vector. Then, linearized vectors were digested with a single endonuclease and transfected into the Y1HGold strain; the strains generated were used as bait. The CDS of *VqMYB154* was integrated into pGADT7 to form AD-VqMYB154. The fusion vector was transfected into baits separately; the pGADT7 vector was also transfected into baits as a control. Transformants were grown on medium with SD/ − Leu with AbA.

### *Agrobacterium*-mediated transient overexpression in grape leaves

The fusion vector 35S-VqMYB154-GFP and empty vector were ligated into *Agrobacterium tumefaciens* GV3101. The transformed strains were grown in a lysogeny broth (LB) liquid medium at 28 °C. After centrifugation, the pelleted bacteria were resuspended (OD_600_ = 0.6). Leaves of Danfeng-2 were immersed in a jar containing an *Agrobacterium* suspension. After vacuum infiltration for 30 min using a previously described method^76^, the samples were stored with the petioles wrapped in moist medical absorbent cotton in trays for 48 h before collection (Supplementary Fig. S2).

### GUS activity analysis

The promoters of *VqMYB154* and *VvMYB154* were ligated into the pC0390-GUS vector, and the fusion vector was infused into GV3101 for transient expression in grape leaves^77^. After vacuum infiltration, the grape leaves were cultivated for 2 days and then infected with *U. necator* for one d before collection. GUS activity experiments were performed as previously described^72^. The CaMV35S-GUS vector was used as the positive control, and the negative control was the pC0390-GUS vector. For the stilbene regulation assay, the *VqSTS* promoters were integrated into the pC1391-GUS vector and then infused into the GV3101 strain. The vector 35S-VqMYB154-GFP was also infused into the GV3101 strain. Strains carrying various vector combinations were infiltrated into tobacco leaves based on *Agrobacterium*-mediated transient transformation^78^. After 72 h of cultivation, GUS activity was detected. The empty vector pC1391-GUS was used as a negative control. A TECAN Infinite M200 PRO Absorbance Microplate Reader (TECAN, Switzerland) was used in the above assays.

### Stilbenoid extraction and analysis

The fully ground powder of grape leaves was dried at −105 °C for 24 h; the samples were weighed and then extracted in methanol (Tedia, Fairfield, USA) away from light at 4 °C overnight. The insoluble solid was discarded by low-temperature centrifugation at 4000 × *g* for 15 min. The clear methanol extracts were filtered through a 0.22 µm membrane film and stored in sample bottles. HPLC determination was conducted using an Agilent 1260 Infinity HPLC system (Agilent, USA). Stilbene was separated from the filtered samples (10 µL) using a binary gradient of solvent A (acetonitrile) and solvent B (ultrapure water). The wavelength for fluorimetric determination was 306 nm. The gradient conditions were based on a previous study^79^. The retention times were confirmed using standard samples of trans-resveratrol and trans-piceid (Sigma-Aldrich, USA). The stilbenoid concentration was determined based on the peak area.

### *Arabidopsis* transformation and disease assays

The GV3101 strain carrying the 35S-VqMYB154-GFP construct was used for *Arabidopsis* transformation. T3 transgenic lines were adopted for disease assays. Four-week-old *Arabidopsis* leaves were infected with a suspension containing *Pst* DC3000 following a previously described method^80^. The samples were used for counting bacterial colonies after inoculation for 3 days. Leaves were acquired at 0, 24, 48, and 72 h postinfection and then used for quantitative RT-PCR. Callose deposition was observed using aniline blue. The transparent leaves decolorized with 95% ethanol were stained with aniline blue solution for 24 h and then observed under a fluorescence microscope (Olympus BX63, Tokyo, Japan) with UV irradiation. Cell death was detected using a trypan blue solution. The samples at 72 h postinfection were submerged in the solution and boiled for staining. The stained leaves were decolorized with chloral hydrate. DAB staining was performed to visualize levels of H_2_O_2_. Leaves to be observed were immersed in DAB, stained for 8 h, and then boiled in 95% ethanol for decolorization. NBT staining was used to visualize O^2−^ levels in the leaves. Leaves at 72 h postinoculation were submerged in NBT solution for 2 h, soaked in 80% ethanol, and decolorized at 60 °C for 2 h.

To perform phenotypic analysis of *Arabidopsis* plants under artificial inoculation with *Golovinomyces cichoracearum*, the *G*. *cichoracearum* isolate UCSC1 was cultivated on *pad4 Arabidopsis* mutants. Four-week-old leaves were used for inoculation according to a previous method^81^. After inoculation for 7 days, the plants were used for phenotypic analysis.

### Measurement of plant physiological indexes

Relative electrolyte leakage (REL) was analyzed following a previous method^82^. Approximately 0.1 g fresh leaves were immersed in 10 mL deionized water. After vacuum infiltration for 20 min, the leaves were allowed to stand for 3 h, and electrical conductivity (EC1) was recorded by a conductivity meter. Next, tubes containing leaf samples were boiled for 20 min and cooled down. The second electrical conductivity (EC2) was then recorded, and the REL ratio was determined (EC1/EC2 × 100%). The total chlorophyll content and malondialdehyde (MDA) content were analyzed as previously described^83,84^. The assimilation rate (A), which represents the net photosynthetic rate, was determined using a portable photosynthetic apparatus CIRAS-3 (PP Systems, USA). A commercial detection kit was used to determine H_2_O_2_ content (Suzhou Keming Bioengineering Institute, China).

### Gene expression analysis by qRT-PCR

RNA from grapes and *Arabidopsis* was extracted according to the manufacturer’s instructions (Omega, Norcross, GA, USA). cDNA was acquired using the FastKing RT kit (Tiangen, Beijing, China). The sample mixture consisted of 7 μL sterile water, 0.8 μL each primer, 1 μL template, and 10 μL SYBR Taq (Takara, Japan). The 2^−∆∆Ct^ method was used for calculations. Grapevine *GAPDH* (XM_002278316.4) and *Arabidopsis Actin* (AT3G18780) were used as standard controls. The assays were performed using Applied Biosystems QuantStudio 6 Flex System (Applied Biosystems, Foster City, USA). Data are presented as the mean (±SD) from three biological replicates. For assays with multiple timepoint controls, the fold change was calculated based on the rate between the treatment group and the mock control at the same timepoint. For screening assays of resistance-related MYB TFs, the fold change was acquired by comparison with the expression in inoculated samples at 0 h. The fold change of gene expression in grapevine organs was calculated by comparison with the expression level in stems. Significance analysis was conducted with GraphPad Prism 7.0 (GraphPad Software, La Jolla, USA) using one-way ANOVA with Fisher’s LSD test (*P < 0.05; **P < 0.01). The qRT-PCR primers used are listed in Supplementary Tables S1 and S2.

## Supplementary information

Supplementary Information
