# Supplementary material for: VqMYB154 promotes polygene expression and enhances resistance to pathogens in Chinese wild grapevine
Source: Hortic Res. 2021 Jul 1;8:151. doi: 10.1038/s41438-021-00585-0 (PMC8245564; doi:10.1038/s41438-021-00585-0)
Supplement: Supplementary file 1 — Supplementary Information [file 41438_2021_585_MOESM1_ESM.pdf]

Supplementary Information

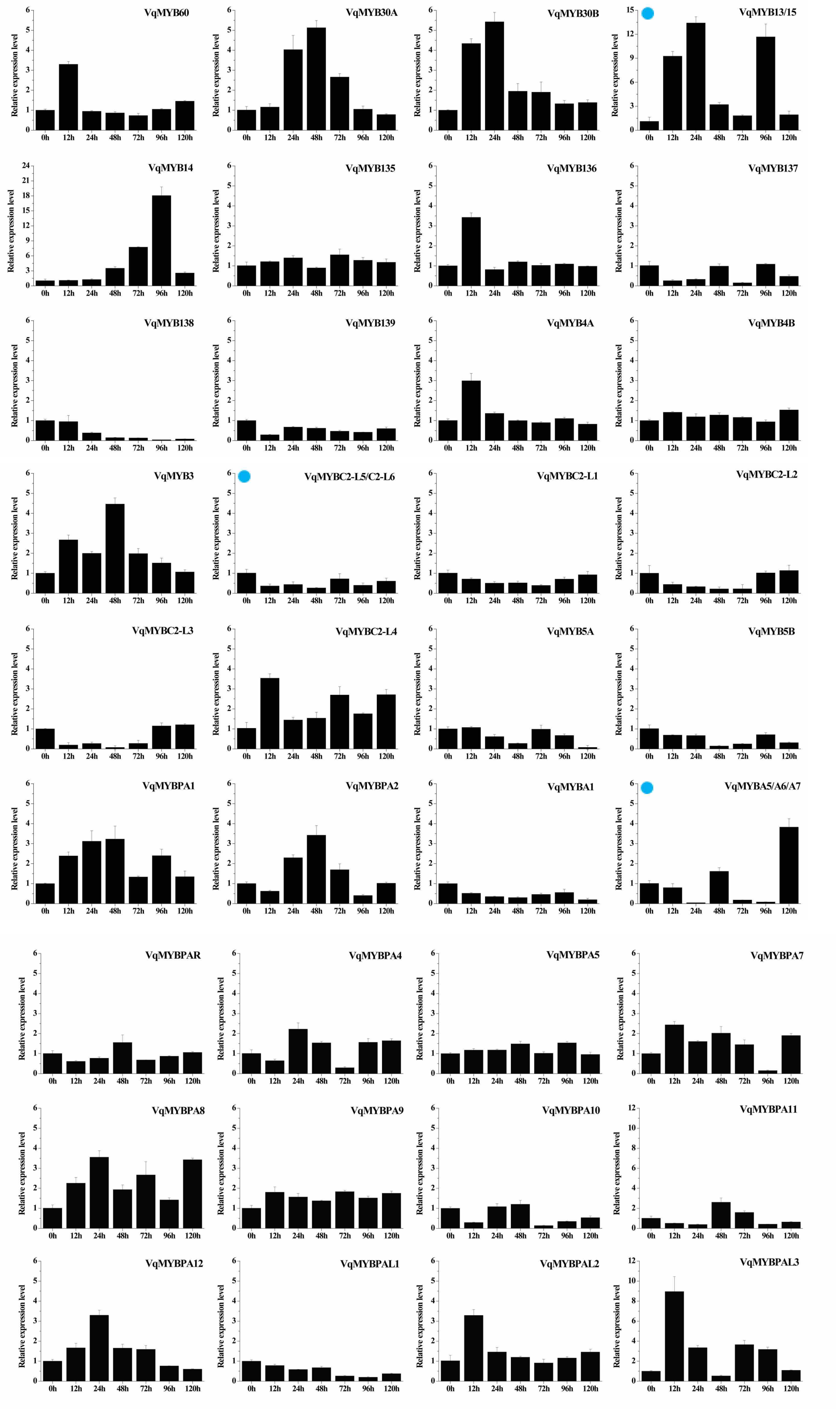

(Fig. S1 to continue)

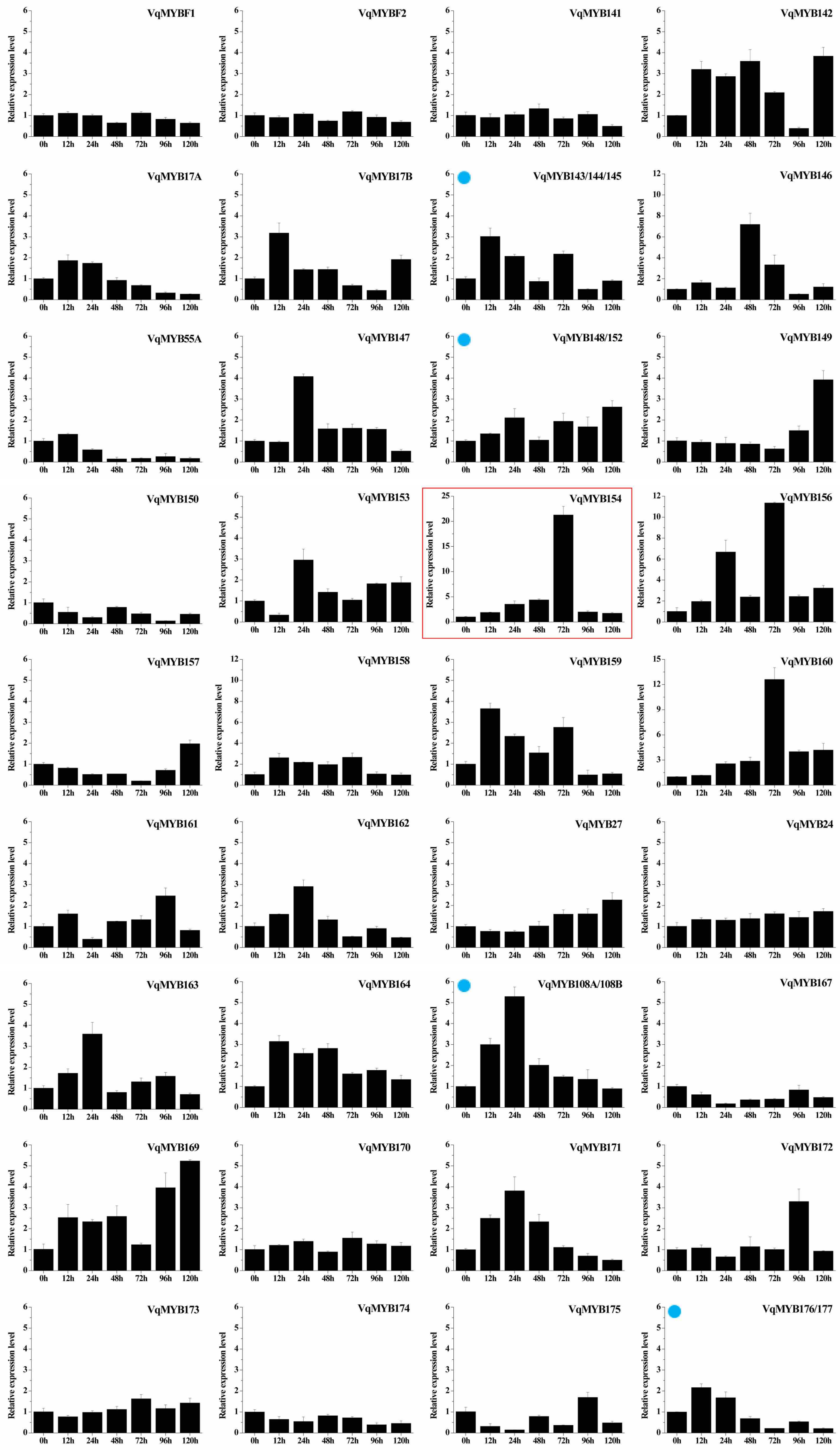

(Fig. S1 to continue)

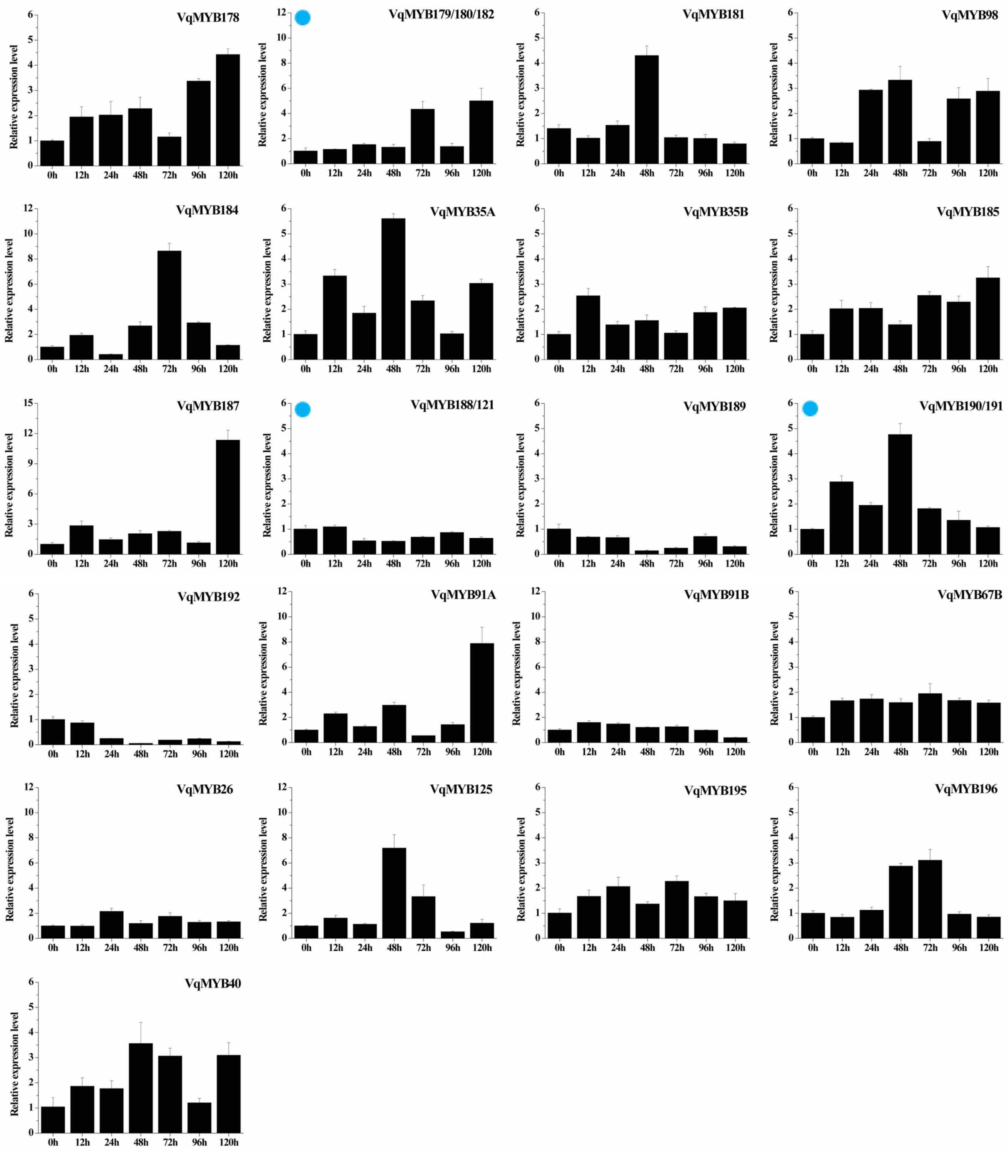

Supplementary Fig. S1 Characteristic expression analyses of 106 R2R3-*MYB* genes in the leaves of Danfeng-2 under artificial inoculation with *Uncinula necator*. The *MYB* genes with high homology were determined using primers based on the sequence of their conservative region and these results are marked with a blue solid circle. *VqMYB154* is marked with a red box. Grape *GAPDH* was used as a reference gene. Data are means (±SD) of three biological replicates.

a

b

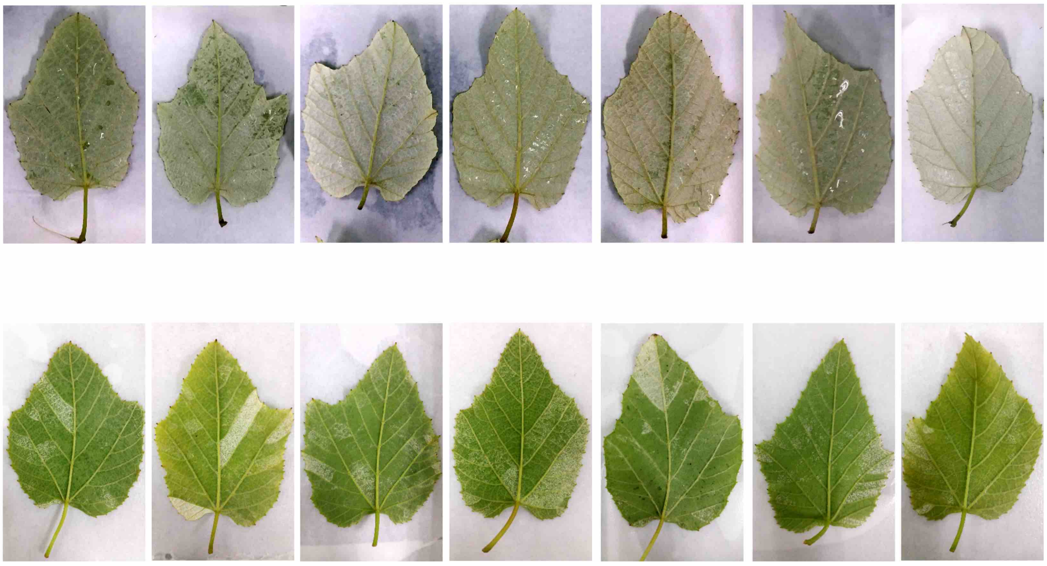

Supplementary Fig. S2 *Agrobacterium*-mediated transient transformation in grape leaves. a Grape leaves before transient transformation. **b** Grape leaves after transient transformation. The sampling position is the area where the *Agrobacterium* suspension liquid has completely penetrated.

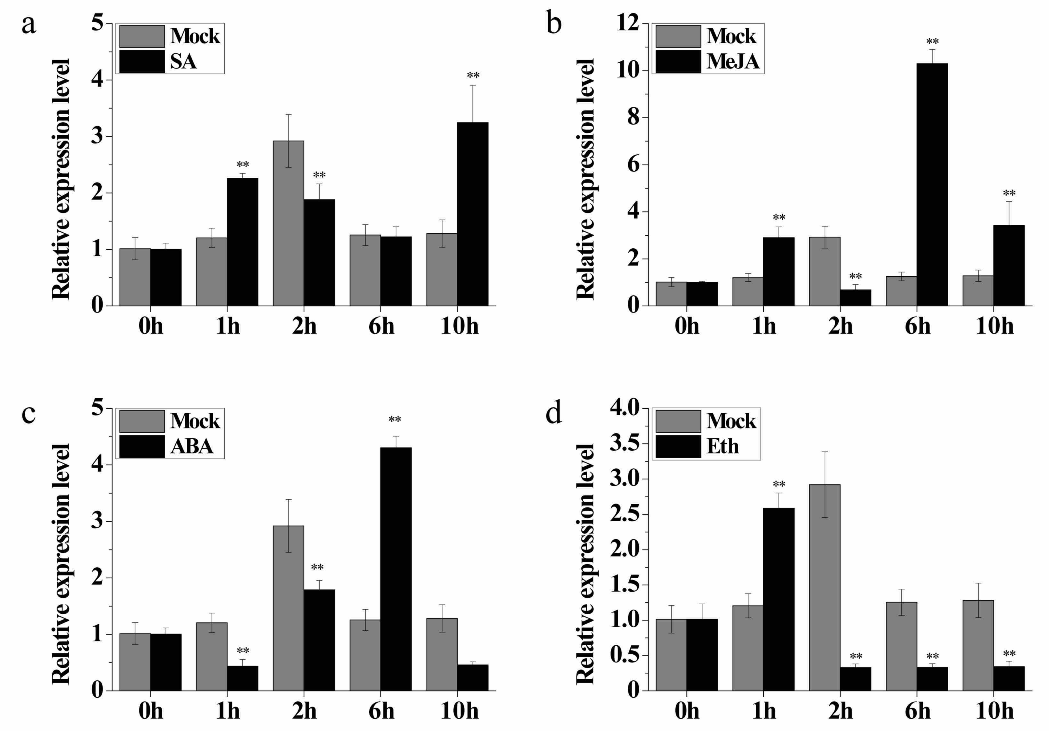

Supplementary Fig. S3 Expression analysis of *VvMYB154* from Cabernet Sauvignon using exogenous phytohormones. Leaves from Cabernet Sauvignon were treated with 100 μM SA **(a)**, MeJA **(b)**, ABA **(c)** and Eth **(d)** and acquired at 0, 1, 2, 6, and 10 h after spraying. Results are shown as the means (±SD) of three biological assays. Significance was determined with GraphPad Prism using a one-way ANOVA with Fisher's LSD test (*P<0.05; **P<0.01).

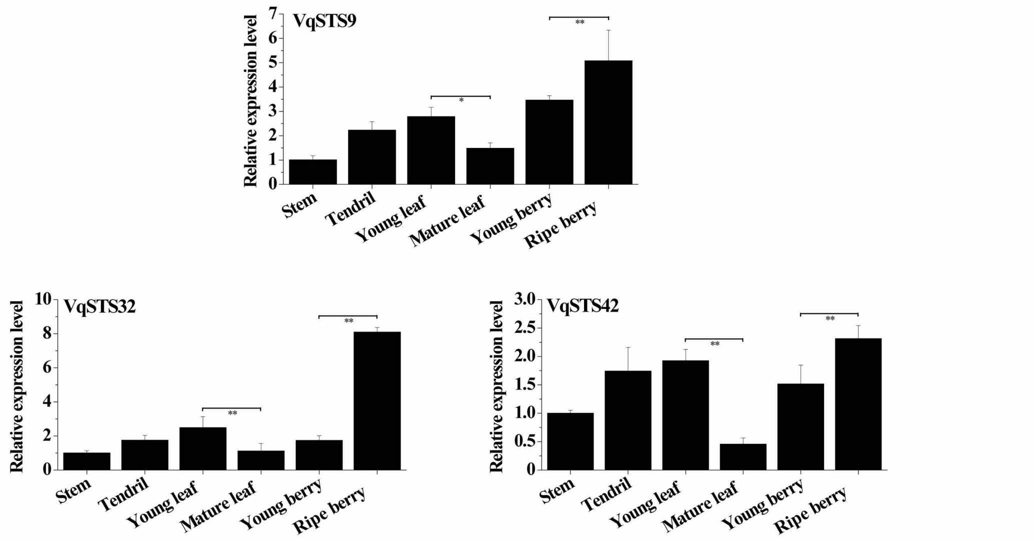

Supplementary Fig. S4 Differential expression of *VqSTS* genes in various organs from Danfeng-2 under natural conditions. The expression patterns of *VqSTS9*, *VqSTS32* and *VqSTS42* in various tissues were determined by qRT-PCR. Results are shown as the means (±SD) of three biological assays. Significance was determined with GraphPad Prism using a one-way ANOVA with Fisher's LSD test (*P<0.05; **P<0.01).

Supplementary Table S1. Sequences of primers used in this study.

Table S1<br>Primer name	List of primer used in this study<br>Primers(5'-3')	Use
VqMYB154-F	ATGGGTAGAGCTCCTTGTTGTGACAAG	Gene clone
VqMYB154-R	AATTTGATTGACCAGACCAGCTTCATG	
RT-VqGAPDH-F	TTCTCGTTGAGGGCTATTCCA	RT-PCR
RT-VqGAPDH-R	CCACAGACTTCATCGGTGACA	
RT-VqMYB154-F	CTGGATTGCTCTTCCTCGTAAA	
RT-VqMYB154-R	TGTTGTCTTCGTCCTCAGTAAAG	
RT-VqSTS32-F	AATGCTCAGCGTGCCAAGGG	
RT-VqSTS32-R	CTCGGTCATGTGCTCGCTCT	
RT-VqSTS42-F	CGCTAAGGATCTTGCAGAGAATA	
RT-VqSTS42-R	AAGGGCTTGGCCAACTAAA	
RT-VqSTS9-F	GGACGAGATGAGGAAGAAATCC	
RT-VqSTS9-R	ACAACAGTCTCGATGGTCAAG	
RT-VqPAL-F	GTTGTCGTGAAAAACCAGCTT	
RT-VqPAL-R	GGATCACTCACGACGAAACTC	
RT-VqSTSs-F	CAAGCCCTTTTTGGTGATG	
RT-VqSTSs-R	CCACAAGTGAAAGGTGAGTCC	
RT-VqRSGT-F	CTACCATGGCTTAGTCCCTTTC	
RT-VqRSGT-R	AGCTGGCGATTTCATCATACT	
RT-VqCHS-F	CATCACAAATAGCGAACACAAG	
RT-VqCHS-R	CCTAGCATCCAGGGAAGC	
RT-AtActin-F	AGTGTCTGGATCGGTGGTTC	
RT-AtActin-R	CCCCAGCTTTTTAAGCCTTT	
RT-AtRBOHC-F	AATTCTCTCATTTTCTCGGG	
RT-AtRBOHC-R	CCACCTTCCATTTTCAAGC	
RT-AtPR5-F	AACGGTAGATGTGTAACCGGAG	
RT-AtPR5-R	CGATCCTCCGGATGGTCTTATC	
RT-AtICS1-F	AATCTGGTTAGCGTTGCTGGTA	
RT-AtICS1-R	CAACAGCGATCTTGCCATTAGG	
RT-AtLOX3-F	AGACAACAGCCGTCGATTTG	
RT-AtLOX3-R	ACGTAACACCAGGCTCAGAA	

(Table S1 to continue)

Table S1<br>Primer name	List of primer used in this study<br>Primers(5’-3’)	Use
RT-AtPDF1.2-F	TCCATCATCACCCTTATCTTCG	
RT-AtPDF1.2-R	GCACTGATTCTTGCATGCATTACTG	
ProVqMYB154-GUS-F-Sal I	CAAGCTTGGCTGCAGGTCGACTAGTATAGAAGACAATAACAACAATGAGTATGAGC	pC0390
ProVqMYB154-GUS-R-BamH I	GGTCTTAGAATTCCCGGATCCCATGTCCACAAACTGAATTGAAACTTACAACAAAC	
VqMYB154-GFP-F-BamH I	GAGCTCGGTACCCGGGGATCCATGGGTAGAGCTCCTTGTTGTGACAAG	pC2300
VqMYB154-GFP-R-Sal I	CTTGCTCACCATGGTGTCGACAATTTGATTGACCAGACCAGCTTCATG	
ProVqSTS9-ABAi-F-Sma I	TCCCCCGGGAGCCTACCAAAGTCAAGTTGTTTCGTGGA	pAbAi
ProVqSTS9-ABAi-R-Xho I	CCCTCGAGTTGATCCTAGCTACGAACTCAAATTGAAGCTG	
ProVqSTS32-ABAi-F-Sma I	CCCCCGGGCCTCTAACTGGAAATCAATCCCTTGTTCATTTG	
ProVqSTS32-ABAi-R-Xho I	CCCTCGAGGCTGTGTCCCACCAACTCTCATCCA	
ProVqSTS42-ABAi-F-Kpn I	GGGTACCCCCACCCGTGGAAAAGTCAAATGAAC	
ProVqSTS42-ABAi-R-Xho I	CCTCGAGGGATGCCAGCTAGGTACTCAAAT	
3×L5box-ABAi-F-Sac I	CGAGCTCACCAACTACCAACTACCAACT	
3×L5box-ABAi-R-Xho I	CCCTCGAGAGTTGGTAGTTGGTAGTTGGT	
3×ACbox-ABAi-F-Sac I	CGAGCTCGAGTTGGTGAGAGAGTTGGTGAGAGAGTTGGTGAGA	
3×ACbox-ABAi-R-Xho I	CCCTCGAGTCTCACCAACTCTCTCACCAACTCTCTCACCAACTC	
3×MYBCORE-ABAi-F-Sac I	CGAGCTCCAGTTACAGTTACAGTTA	
3×MYBCORE-ABAi-R-Xho I	CCCTCGAGTAACTGTAACTGTAACTG	
ProVqSTS9-GUS-F-BamH I	CGGGATCCAGCCTACCAAAGTCAAGTTGTTTCGTGGA	pC1391
ProVqSTS9-GUS-R-Sma I	CCCCCGGGTTGATCCTAGCTACGAACTCAAATTGAAGCTG	
ProVqSTS32-GUS-F-BamH I	CGGGATCCCCTCTAACTGGAAATCAATCCCTTGTTCATTTG	
ProVqSTS32-GUS-R-Sma I	CCCCCGGGGCTGTGTCCCACCAACTCTCATCCA	
ProVqSTS42-GUS-F-BamH I	CGGGATCCCCCACCCGTGGAAAAGTCAAATGAAC	
ProVqSTS42-GUS-R-Sma I	CCCCCGGGGGATGCCAGCTAGGTACTCAAAT	
VqMYB154-AD-F-Cla I	CCATCGATACATGGGTAGAGCTCCTTGTTGTGACAAG	pGADT7
VqMYB154-AD-R-Xho I	CCCTCGAGCTAAATTTGATTGACCAGACCAGCTTCATG	
VqMYB14-AD-F-EcoR I	CGGAATTCATGGGGAGAGCTCCATGTTGTG	
VqMYB14-AD-R-BamH I	CGGGATCCTCATATTTCTGATAATTCATGCAACTCCC	
VqMYB15-AD-F-EcoR I	CGGAATTCATGGTAAGAGCTCCTTGTTGTGATAAG	
VqMYB15-AD-R-BamH I	CGGGATCCTCAAAGCTCCTGTAAGCCGCC	

(Table S1 to continue)

**Table S1**<br>**Primer name**	**List of primer used in this study**<br>**Primers(5'-3')**	**Use**
VqMYB154-BD-F-Sal I	ACGCGTCGACCTATGGGTAGAGCTCCTTGTTGTGACAAG	pGBKT7
VqMYB154-BD-R-Pst I	AACTGCAGCTAAATTTGATTGACCAGACCAGCTTCATG	

Supplementary Table S2. Primers used in qRT-PCR for characteristic expression detection of resistance to *Uncinula necator* among 106 *VqMYBs* from *Vitis quinquangularis* accession Danfeng-2.

Table S2<br>Primer name	Primer sequence<br>Primers(5'-3')
RT-VqMYB60-F	CAAGGCTACTAGAAGGTTGGATG
RT-VqMYB60-R	CCATGGAGTTTCCGGTCATATC
RT-VqMYB30A-F	GAAGTTTCCCACAGGTGTAGAT
RT-VqMYB30A-R	TAGCCATGTGGATGTCTGTTT
RT-VqMYB30B-F	GATTCGACTCCTTGGACTCTTC
RT-VqMYB30B-R	AGCACCGCCTTCTTCAAATA
RT-VqMYB13/15-F	GGTCGGACTGACAATGAGATAAA
RT-VqMYB13/15-R	GCAGTAGAGTGGCCTTTAGAAT
RT-VqMYB14-F	TCTGAGGCCGGATATCAAAC
RT-VqMYB14-R	GGGACGCATCAAGAGAGTGT
RT-VqMYB135-F	AGGCTTCGATGGACGAACTA
RT-VqMYB135-R	CCAAGAAGTTGATGGAGCTTGA
RT-VqMYB136-F	ACTGTTGAAGCTAAGCCGATAG
RT-VqMYB136-R	GACTGGTGATTGATGAAGAGGAG
RT-VqMYB137-F	CCAGGAAGAACAGACAATGAGA
RT-VqMYB137-R	GGAGAGACCATTGGAGATGAAG
RT-VqMYB138-F	CGCCAGCTGTGAATGAAATG
RT-VqMYB138-R	GGGTGTAGGAATCGTCTGTTAAG
RT-VqMYB139-F	CCTAGGCTCATTCCAGTACAAC
RT-VqMYB139-R	TGGGCTGCATCTCCATTATC
RT-VqMYB4A-F	ACCGGACGTTACAACCATATC
RT-VqMYB4A-R	GGTTGAGGTCTGGACACTTT
RT-VqMYB4B-F	GCCGAAACCCAGATGAAGAA
RT-VqMYB4B-R	CCAGGCTGCAGTAGAAACAA
RT-VqMYB3-F	GTCACCACTGAAGAACTCTACC
RT-VqMYB3-R	CGGTTGTTTCTTTGGTTCCTTTA
RT-VqMYBC2L5/L6-F	CTCATCAGTGGCGGAATCAA
RT-VqMYBC2L5/L6-R	TGTGGTGGTGATGGTCAAAG
RT-VqMYBC2L1-F	TGATGCCGGAAGCTGTTTAG

(Table S2 to continue)

**Table S2**<br>**Primer name**	**Primer sequence**<br>**Primers(5'-3')**
RT-VqMYBC2L1-R	CGCCTCTTCGGTAGTATCTAGT
RT-VqMYBC2L2-F	GCTTCGGGTACTAGCAAATCA
RT-VqMYBC2L2-R	CGCTGAGCCACATGTAGAAT
RT-VqMYBC2L3-F	ACTGGCGCATGCGATAATA
RT-VqMYBC2L3-R	GTCAAGGGAGGCGGATATTT
RT-VqMYBC2L4-F	TCCCTGATAGCGGGTAGATT
RT-VqMYBC2L4-R	GATACCCATCCTTATGAGCTTTCT
RT-VqMYB5A-F	CTAGAACTGTCTGGGAACCT
RT-VqMYB5A-R	TGCAAGGATCCATTTCACATAC
RT-VqMYB5B-F	TGACAGCCGGTGTTCTTTAAT
RT-VqMYB5B-R	AGCATACTAACACAACAACACAACC
RT-VqMYBPA1-F	AGATCAACTGGTTATGCTTGCT
RT-VqMYBPA1-R	AACACAAATGTACATCGCACAC
RT-VqMYBPA2-F	CCTCTGATGCTCTCAACTCATT
RT-VqMYBPA2-R	GAAGACCACTCACTCCTTCTTG
RT-VqMYBA1-F	GAGGGTGATTTTCCATTTGAT
RT-VqMYBA1-R	CAAGAACAACTTTTGAACTTAAACAT
RT-VqMYBA5/A6/A7-F	ACTTGTCCTCGGACCTTCTA
RT-VqMYBA5/A6/A7-R	GACAGAGGTGGCGTTGAATA
RT-VqMYBPA4-F	ACTCTTTGTCCTCCGGATTTC
RT-VqMYBPA4-R	CGCCCACCACGTTATTATCT
RT-VqMYBPAR-F	GCCGACAGGAACAAGAACA
RT-VqMYBPAR-R	CTGCCAGTGGAGCTATGAATAC
RT-VqMYBPA5-F	GCCGAATTCCACCTCGATTA
RT-VqMYBPA5-R	TGGCTCAGTGGCAATGAATA
RT-VqMYBPA7-F	GAAGATAAGATACTCACGGCTTACA
RT-VqMYBPA7-R	CAGCCATCTCAGTCTACAACTC
RT-VqMYBPA11-F	CCAAAGCAAAGACGACAGAAAG
RT-VqMYBPA11-R	CATTGCTGGGTTTGGTGAATAA
RT-VqMYBPA8-F	CAGGGCGAACAGACAATGA

(Table S2 to continue)

Table S2<br>Primer name	Primer sequence<br>Primers(5’-3’)
RT-VqMYBPA8-R	AGCAAATCTGATGGAGGAGAAG
RT-VqMYBPA9-F	GGACAGCTCTTGAGGACAAA
RT-VqMYBPA9-R	ACCATCTAAGCCTGCAACTC
RT-VqMYBPA10-F	CCTCTACTGTTTCCTGTTGATGA
RT-VqMYBPA10-R	CTGGTGGTTGTTCTGCTACTAT
RT-VqMYBPA12-F	TTCGGACCAAGGCATTTAGG
RT-VqMYBPA12-R	GGTTCATCATGGAGGGTTCTT
RT-VqMYBPAL1-F	GAGGATGGCTTATGTGAGAGTG
RT-VqMYBPAL1-R	AACTGCTGTCCACCATCTTC
RT-VqMYBPAL2-F	CAAAGGTGATGGAGATGGAGAG
RT-VqMYBPAL2-R	CAAAGGGCCCTCACTAGAATAA
RT-VqMYBPAL3-F	TGCCAGGTCGAACAGATAATG
RT-VqMYBPAL3-R	AGTTTGGAGCAGCCTCTTG
RT-VqMYBF1-F	CGATGATGATGGGAATGGAGTAA
RT-VqMYBF1-R	CCAGAAGATGACTCGCCATAAG
RT-VqMYBF2-F	GGGAGTGGTTTACTCCTTCATC
RT-VqMYBF2-R	CCTCCATCCCAAAGCTCATAG
RT-VqMYB141-F	GTTCTCCTACTTCGACGCTTAG
RT-VqMYB141-R	CATGCCTTTCCACTCTTGTTG
RT-VqMYB142-F	GGCACGTTAGGGACTGAAAT
RT-VqMYB142-R	CCGTCACCAGACCTACTAAGA
RT-VqMYB17A-F	CAACGAGCTGGAGGATTCAT
RT-VqMYB17A-R	CATAGCAGGGAATGTGGGATAG
RT-VqMYB17B-F	GGACCAGCACATCTACATCAA
RT-VqMYB17B-R	CTGAACCGGTCATCATGTCTT
RT-VqMYB143/144/145-F	GGAAGGACCGACAACGAAAT
RT-VqMYB143/144/145-R	CAGAAGATCGAGTCGAGGACTA
RT-VqMYB146-F	GCTAGAGCTAGAGCCACTACTA
RT-VqMYB146-R	GATCCGGGATGCAGATCAAA
RT-VqMYB55A-F	ATCCAGTGAATTGAACCTCCTC

(Table S2 to continue)

**Table S2** **Primer name**	**Primer sequence** **Primers(5’-3’)**
RT-VqMYB55A-R	CCATTCCATAGCCTCGTGTT
RT-VqMYB147-F	GACTTCCTGCAGTACCATCATC
RT-VqMYB147-R	GCACTCTTGACCCTTCTGTTAG
RT-VqMYB148/152-F	CGTTGGCAGAAACCCTTATTG
RT-VqMYB148/152-R	ATGGATGCTTGGTCCTTGAT
RT-VqMYB149-F	GTACTTTCCCAGGCCATGAA
RT-VqMYB149-R	CTCTTGGGTTTGAAGGAGAGAG
RT-VqMYB150-F	GAGCCGGAGTATGATCAGTATG
RT-VqMYB150-R	CGCCACCTCCACTACTATTT
RT-VqMYB153-F	GGCTTCTGGTTTCAGGAGAA
RT-VqMYB153-R	CCTTGTGGCTTAATGTTGAGATG
RT-VqMYB154-F	AGAGTTGCAGGTTGAGATGG
RT-VqMYB154-R	TGTTGTTGTAGAGAGTGACGATAA
RT-VqMYB156-F	TTCTTGTGGAAGCAGCAGAG
RT-VqMYB156-R	ACCAGTTGATCATGCCAGTC
RT-VqMYB157-F	ACCCAGATTCTAAACCACCTTC
RT-VqMYB157-R	CTCTGTGCCCATTTCCATATCT
RT-VqMYB158-F	CGGCCAGCTCTTTGTTAATTC
RT-VqMYB158-R	GACTCCTCGTTCAGTAGGAAAC
RT-VqMYB159-F	CCCAAACCAGATGGACTTATCC
RT-VqMYB159-R	CATCTCTCCAGGTAGCTTGTTC
RT-VqMYB160-F	CCAACAACAATGCCGGTTATAC
RT-VqMYB160-R	AACTGGAGACTGGGAGAGAA
RT-VqMYB161-F	AGTCCTTTGGGTTGCCTTATC
RT-VqMYB161-R	GCCCAGAAATGGGCTTAGAA
RT-VqMYB162-F	AATCTCCCTGTCCTCCATCT
RT-VqMYB162-R	TGGGCTGAAAGAGTAGTGATTG
RT-VqMYB24-F	ATTCTCCACCATCCTACCCT
RT-VqMYB24-R	GTAGCTGCATAGACCAGATGTC
RT-VqMYB163-F	CTGCTGCTGATACTGTGACTAC

(Table S2 to continue)

Table S2<br>Primer name	Primer sequence<br>Primers(5'-3')
RT-VqMYB163-R	GATGAGACAGTGATCTGGTTGG
RT-VqMYB164-F	TGATTCGGTGTGTGTGGATG
RT-VqMYB164-R	TCGGCATGGTTGAGAGATTG
RT-VqMYB108A/B-F	ATGAACATGGACCTGAGGAAAG
RT-VqMYB108A/B-R	TTCGCCGTGGATGGTAATG
RT-VqMYB167-F	CTTCATCTGGCACTGATCTCTC
RT-VqMYB167-R	CTGCTGCATAGCAGGAGTATAA
RT-VqMYB169-F	GCAAGGAGAGATGATGAAGAGG
RT-VqMYB169-R	TCAAGAAGAGCTTCCAACTGATAG
RT-VqMYB170-F	CTTCGGCATATGGCGGATATAG
RT-VqMYB170-R	CACCGAGTTTAGAGTACCCAAC
RT-VqMYB171-F	GGCTTTCCCGTAGTTTCTTCT
RT-VqMYB171-R	CGTTGGAGGATCGTCTTTCTT
RT-VqMYB172-F	ACTTCTCTCTCCCTCTCTCTTC
RT-VqMYB172-R	CAAAGGAGCCATCGTAGGTATC
RT-VqMYB173-F	CAACAGCTGCGATAGGGTTA
RT-VqMYB173-R	TCGAGCATCCTCCACTCTAT
RT-VqMYB174-F	CTGTGAGTTGGGTTTAGGTATGT
RT-VqMYB174-R	CCGACATGTAGTTCCTCACTTC
RT-VqMYB175-F	GGATGCGATGAGAGGTGTTATAG
RT-VqMYB175-R	CACAGAAAGTGTGAGGAAGGT
RT-VqMYB176/177-F	GCAGAACAGACAATGCAATCAA
RT-VqMYB176/177-R	GGGCTACCATCTTCCAACATAC
RT-VqMYB178-F	CTCGAGCAGTTCCTACGATATTC
RT-VqMYB178-R	AGATGCAAGGCAGGTTCAT
RT-VqMYB179/180/182-F	CTGGTTAACCTGAAGGAGCTTAT
RT-VqMYB179/180/182-R	GCTGGAGATTGAAGGAGATACTG
RT-VqMYB181-F	ACCTCTTTGGAACACCATCTT
RT-VqMYB181-R	CTGTTGGGTGGAGACCAATTA
RT-VqMYB98-F	GCTCCTACTGGTGATGAGAAAG

(Table S2 to continue)

**Table S2**<br>**Primer name**	**Primer sequence**<br>**Primers(5'-3')**
RT-VqMYB98-R	CCACCAAATCCATCTCCTTCTT
RT-VqMYB184-F	TTGAGGGCTCTTCCATGAATC
RT-VqMYB184-R	GCGCCATAAGTACTGGTATCTC
RT-VqMYB35A-F	CAACACCGCAACACGAATG
RT-VqMYB35A-R	TGAGAACCTGGAATTGCTCTAC
RT-VqMYB35B-F	CCAAAGAATGGAACCCGAATTG
RT-VqMYB35B-R	GGCTTTAAGCTGAGTGAGAAGA
RT-VqMYB185-F	GGATCATGTTGGGCTCCTT
RT-VqMYB185-R	CGCCCATGCTTATACTCTCTAC
RT-VqMYB27-F	TCTTCGCCCTGACCTTAAAC
RT-VqMYB27-R	ATCCACGACCACTTGTTACC
RT-VqMYB187-F	TCTCCGGTGTGGGACTATT
RT-VqMYB187-R	ACGCCATTCCGTGTTCAT
RT-VqMYB188/121-F	GAACTACTGGAGGACCCATTTC
RT-VqMYB188/121-R	TGCTGCTGCTGCTGATAAA
RT-VqMYB40-F	CTTGGAGTTGGGACTACTGATG
RT-VqMYB40-R	CCATGGAAGGGTTGAGAGAAT
RT-VqMYB189-F	CTTCTTCATCTGCTTCGTCCA
RT-VqMYB189-R	TCATCCCAGAAGCCCAAATC
RT-VqMYB190/191-F	GGTTGCCTGGAAGAACAGATA
RT-VqMYB190/191-R	GACTGGGTCAATTCCCATCTT
RT-VqMYB192-F	AAGCACAGAGTTCAGTTCCC
RT-VqMYB192-R	CCATTCCAAGTGCTCTCAGTAG
RT-VqMYB91A-F	GCAGAGAGAGCAGAAAGAGAAC
RT-VqMYB91A-R	GCAGGACGCTCTTTCACTAA
RT-VqMYB91B-F	TGATGCCGGTCCAACAAA
RT-VqMYB91B-R	CTCCATGTCGCTTCCTTCTT
RT-VqMYB67B-F	CATTGGAAGCACACCCATTATTT
RT-VqMYB67B-R	CAAGAGGTTCAACATGAGCATTC
RT-VqMYB26-F	CTGCCAGATCCTTCTTCGTATG

(Table S2 to continue)

Table S2<br>Primer name	Primer sequence<br>Primers(5'-3')
RT-VqMYB26-R	CTTCATTGCCCAGCACATAATC
RT-VqMYB125-F	TGCCAGGGAGAACTGATAATG
RT-VqMYB125-R	TGGGAAGAAGATGGTGAAGAAG
RT-VqMYB195-F	GGCTTCTACTGCTGCTTATCT
RT-VqMYB195-R	CCTGCTGTACTACTGGGTTATG
RT-VqMYB196-F	AGGAACACCTCATGGATGATAAG
RT-VqMYB196-R	GGATCAACGTTGGGAATCAAAG
